# Green Synthesis and Anticancer Potential of 1,4-Dihydropyridines-Based Triazole Derivatives: In Silico and In Vitro Study

**DOI:** 10.3390/life12040519

**Published:** 2022-03-31

**Authors:** Sabera Bijani, Danish Iqbal, Sheefa Mirza, Vicky Jain, Sadaf Jahan, Mohammed Alsaweed, Yahya Madkhali, Suliman A. Alsagaby, Saeed Banawas, Abdulrahman Algarni, Faris Alrumaihi, Rakesh M. Rawal, Wael Alturaiki, Anamik Shah

**Affiliations:** 1 Department of Chemistry, Marwadi University, Rajkot 360005, Gujarat, India; sabera.bijani@marwadieducation.edu.in (S.B.); vicky.jain@marwadieducation.edu.in (V.J.); 2 Center of Excellence, National Facility for Drug Discovery Complex, Department of Chemistry, Saurashtra University, Rajkot 360005, Gujarat, India; 3 Department of Medical Laboratory Sciences, College of Applied Medical Sciences, Majmaah University, Al Majmaah 11952, Saudi Arabia; s.jahan@mu.edu.sa (S.J.); m.alsaweed@mu.edu.sa (M.A.); y.madkhali@mu.edu.sa (Y.M.); s.alsaqaby@mu.edu.sa (S.A.A.); s.banawas@mu.edu.sa (S.B.); w.alturaiki@mu.edu.sa (W.A.); 4 Health and Basic Sciences Research Center, Majmaah University, Al Majmaah 15341, Saudi Arabia; 5 Department of Internal Medicine, Faculty of Health Sciences, University of the Witwatersrand, Johannesburg 2193, South Africa; mirza.sheefa@yahoo.co.in; 6 Department of Biomedical Sciences, Oregon State University, Corvallis, OR 97331, USA; 7 Department of Medical Laboratory Technology, Faculty of Applied Medical Sciences, Northern Border University, Arar 91431, Saudi Arabia; abdulrahman.eid@nbu.edu.sa; 8 Department of Medical Laboratories, College of Applied Medical Sciences, Qassim University, Buraydah 51425, Saudi Arabia; f_alrumaihi@qu.edu.sa; 9 Department of Life Sciences, Gujarat University, Ahmedabad 380009, Gujarat, India; rakeshrawal@gujaratuniversity.ac.in; 10 B/H Forensic Laboratory, Saurashtra University Karmachari Cooperative Society, Rajkot 360005, Gujarat, India

**Keywords:** click chemistry, green synthesis, cytotoxicity, apoptosis, cheminformatics, protein kinase, tankyrase

## Abstract

A library of 1,4-dihydropyridine-based 1,2,3-triazol derivatives has been designed, synthesized, and evaluated their cytotoxic potential on colorectal adenocarcinoma (Caco-2) cell lines. All compounds were characterized and identified based on their ^1^H and ^13^C NMR (Nuclear Magnetic Resonance) spectroscopic data. Furthermore, molecular docking of best anticancer hits with target proteins (protein kinase CK2α, tankyrase1, and tankyrase2) has been performed. Our results implicated that most of these compounds have significant antiproliferative activity with IC_50_ values between 0.63 ± 0.05 and 5.68 ± 0.14 µM. Moreover, the mechanism of action of most active compounds **13ab′** and **13ad′** suggested that they induce cell death through apoptosis in the late apoptotic phase as well as dead phase, and they could promote cell cycle arrest at the G2/M phase. Furthermore, the molecular docking study illustrated that **13ad′** possesses better binding interaction with the catalytic residues of target proteins involved in cell proliferation and antiapoptotic pathways. Based on our in vitro and in silico study, **13ad′** was found to be a highly effective anti-cancerous compound. The present data indicate that dihydropyridine-linked 1,2,3-triazole conjugates can be generated as potent anticancer agents.

## 1. Introduction

Cancer is an uncontrollable somatic cell proliferation and decline in programmed cell death, which is the subsequent foremost reason of yearly death (nearly 10 million) worldwide among all noncommunicable diseases (41 million) [1]. Colorectal cancer is the second leading cause of death (0.935 million) among all cancer-related deaths globally in 2020 and the third leading cause of cancer cases (1.93 million) [2]. Several proteins such as protein kinase CK2α human tankyrases (TNKS), when overexpressed, were noticed to be engaged in cell proliferation, uncontrollable cell growth, and antiapoptotic role [3,4,5,6,7,8]. A number of cytotoxic agents were developed, but their clinical use is still very limited due to high toxicity or more side effects and the development of resistance to the treatment [9,10].

**Figure 1:**
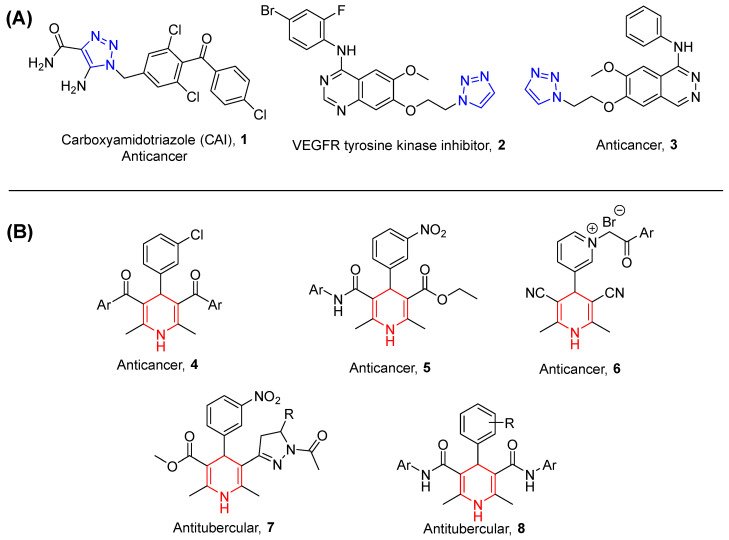
(**A**) Some examples of triazole-containing molecules having anticancer activity. (**B**) 1,4-dihydropyridines-based bioactive molecules.

A survey of literature revealed that N-containing heterocyclic molecules have received much attention during recent years due to their potent biological and pharmacological properties [11,12]. Among them, 1,2,3-triazole moiety has proven its biological significance in the development of potent molecules having various activities such as antioxidant, antimicrobial, antifungal, anti-HIV, anti-inflammatory, antitubercular and anticancer activities (Figure 1A) [13,14,15,16]. This is because of the capability of 1,2,3-triazole to easily bind with various types of enzymes and receptors with different types of bonds [17]. The Huisgen 1,3-dipolar cycloaddition reaction between organic azides and alkynes in the presence of copper (I) catalyst known as click reaction is broadly used in the synthesis of 1,2,3-triazoles [18].

On the other hand, 1,4-dihydropyridines (DHP) are also one of the important N-containing heterocyclic scaffolds that exhibit several biological activities such as calcium channel antagonist, antitumor, anti-inflammatory, antimicrobial, antihistamine, anticonvulsant, and analgesics [19,20,21,22,23]. The distinctive feature of the DHP scaffold is the possibility of structural variations via presenting various chemical substituents in diverse positions of the DHP ring and henceforth providing notable changes in pharmacological profile. In addition to this, with our long-standing interest and experience in the synthesis of 1,4-dihydropyridines-based bioactive molecules (Figure 1B), we observed that 1,4-dihydropyridine-based compounds afford molecules with improved and wide-ranging biological activities such as antitubercular and cancer reversers [24,25,26].

Recently, in the past few years, the synthesis of hybrid bioactive compounds consisting of two or more heterocyclic scaffolds has been focused on more [27]. It has been reported that the combination of two heterocyclic scaffolds having different biological functions produce new hybrid compounds that are more medically effective than their parent molecules [28].

Keeping these observations along with our previous results in view, we aimed to prepare a library of dihydropyridines linked 1,2,3-triazoles and evaluate their therapeutic potential as antiproliferative agents against colorectal cancer Caco-2 cell lines. Moreover, the best hits of compounds were also analyzed for their ability of cell cycle arrest and induction of apoptosis to understand the mechanistic pathways of the antiproliferation activity. Furthermore, a cheminformatics study was performed to predict the inhibitory effects of best hits derivatives against protein kinase CK2α human tankyrases (tankyrase1 and tankyrase2).

## 2. Methodology

### 2.1. Synthetic Procedure for 1,4-Dihydropyridines-Based 1,2,3–Triazole Derivatives

We have synthesized a 1,4-dihydropyridine component by Hantzsch pyridine synthesis methodology in a typical procedure. A solution of substituted propargylated benzaldehyde in glacial acetic acid and 3-amino crotono nitrile was refluxed to get dihydropyridine derivatives [29]. Furthermore, the desired compounds (**13aa′**–**13ag′** and **14ba′**–**14bg′**) were synthesized by the 1,3-diploar cycloaddition reaction between propargylated 1,4-dihydropyridines and substituted azides derivatives under click chemistry conditions in quantitative yields followed by the plausible mechanism as reported earlier [30] with some modification. Briefly, a combination of substituted (hydroxyphenyl)-2,6-dimethyl-1,4-dihydropyridine-3,5-dicarbonitrile 11 (1 equiv.), phenylazide 12 (1.1 equiv.), Cu(OAc)_2_·H_2_O (2 mol%), NH_2_NH_2_·H_2_O (1 mol%), and water was charged into a sealed tube. The reaction was continued under stirring for about one hour at room temperature (27 °C). After accomplishment of the reaction (validated by TLC), the reaction solution was poured into crushed ice and extracted with ethyl acetate to obtain the desired product. Detailed synthetic procedure information with material used has been provided as supplementary information (Section S1. Synthetic Procedure, Figures S1–S14).

### 2.2. Anticancer Evaluation

#### 2.2.1. Cell Culture and Cell Viability Assay

Caco-2 cell line was gifted from Zydus Research Centre, Ahmedabad, Gujarat. Cells were cultured in Dulbecco’s Modified Eagle Medium (DMEM) from Himedia supplemented with 20% Fetal bovine serum (FBS) from Cellclone, and 1% antibiotic (penicillin-streptomycin, Himedia) as described in our previous study [31]. Further, 1 × 10^4^ cells (in 100 µL of media) were seeded in a 96-well plate for 24 h to evaluate the anti-cancerous effect of these synthesized compounds using MTT assay as reported [31,32]. After 24 h, Caco-2 cells were exposed to these synthesized drugs at different concentrations (0.05 to 100 µM) for another 24 h. Stock solutions for all drugs were prepared using dimethyl sulfoxide (DMSO). Then, media was removed, and 100 µL of media containing 10 µL of MTT was added to each well, and plates were incubated for 4 h at 37 °C. A total of 100 µL of DMSO were added to each well after removing the media containing MTT to dissolve the purple-colored formazan crystals. Using ELISA reader (Multiskan Spectrum Microplate Reader, Thermo Scientific, Waltham, MA, USA), optical density was measured at 570 nm wavelength. Along with these synthesized compounds, we have used carboplatin, gemcitabine, and daunorubicine in the range of 0.05 to 50 µM as reference drugs that are widely used in anti-cancerous therapeutic regimens. The untreated cell (without synthesized compounds) was used as negative control, and the growth inhibition (percentage) at various concentrations were calculated by the following equation:

Percentage growth inhibition = 100 − [Mean absorbance of test group/Mean absorbance of control group] × 100

Furthermore, the IC_50_ value (concentration of the inhibitor that inhibits the 50% of cell growth) was calculated through the GraphPad tool/Excel.

#### 2.2.2. Apoptosis/Cell Death Assay

Based on their IC_50_ values, **13ab′** and **13ad′** were selected for further experiments among all synthesized compounds. Cells were exposed to **13ab′** and **13ad′** for 24 h at their IC_50_ values (1.39 ± 0.04 and 0.63 ± 0.05 µM, respectively) to distinguish between apoptotic and necrotic cells using Muse™ Annexin V and Dead Cell Assay kit (Muse™ Cell Analyzer; Millipore, Billerica, MA, USA). After exposing cells to **13ab′** and **13ad′** for 24 h, annexin V and dead cell marker from a kit was used to stain treated and untreated cells as described in the manufacturer’s protocol. Briefly, cells were trypsinized using trypsin-EDTA (Himedia) to get cells in suspension and washed with phosphate buffer saline (PBS) two times. Cells were resuspended in fresh media containing 1% FBS, and from that, 100 µL of cells (1 × 10^5^ cells/mL) were taken. To each tube, 100 µL of Muse Annexin V & Dead Cell Reagent from the kit was added, and tubes were mixed thoroughly and kept in the dark for 20 min to stain cells. Further, results were analyzed using Muse analyzer [33].

#### 2.2.3. Cell Cycle Assay

A cell cycle study was applied to assess the effectiveness of **13ab′** and **13ad′** on cell cycle arrest. The cell lines of Caco-2 (1 × 10^5^ cells/mL) were treated with the respective IC_50_ value concentration (1.39 ± 0.04 and 0.63 ± 0.05 µM, respectively) of **13ab′** and **13ad′** for 24 h to illustrate the impact on cell cycle using Muse^®^ Cell Cycle Assay Kit (Luminex, Austin, TX, USA) according to manufacturer’s instructions as previously described [34]. Cells were collected in a pellet and washed twice with PBS. After that, cells were resuspended in 1 mL of ice-cold 70% ethanol to fix the cells and freeze them for at least 3 h, at −20 °C. Then, 200 µL of fixed cell suspension was taken in a test tube and centrifuged at 300× *g* for 5 min to get the pellet. This pellet was again washed twice with PBS and finally resuspended in 200 µL of Muse™ Cell Cycle Reagent. Tubes were incubated for 30 min in the dark prior to analyzing results on Muse™ Cell Analyzer. The outcomes were recorded in the percentage of cells available in different phases of the cell cycle.

#### 2.2.4. Statistical Analysis

All data from triplicate assay were articulated as mean ± standard error of the mean (SEM), and Student’s *t*-test was used to measure differences among control vs. experimental set. *p* Values < 0.05 were considered statistically significant. All experiments were performed in triplicates.

### 2.3. Cheminformatics Molecular Interaction Study

Autodock-Vina and PyRx tool was used for molecular docking study with the Lamarckian genetic algorithm as scoring function [35,36]. In contrast, the Discovery Studio visualizer 2021 (BIOVIA) tool was used for visualizing the interaction of molecular complex [37].

The X-ray 3-D crystallographic structure of target proteins, namely protein kinase CK2 (PDB Id: 3PE1) at 1.60 Å resolution, human tankyrase1 (TNKS1) (PDB Id: 4W6E) at 1.95 Å resolution, and human tankyrase2 (TNKS2) (PDB Id: 4HKI) at 2.15 Å resolution in complex to their native inhibitors were obtained from PDB (Protein Data Bank) database [38,39,40]. The heteroatoms were removed from proteins, and adding of polar hydrogens was performed to prepare it for molecular docking work, thereafter converting the “.pdb” format file to Autodock suitable “.pdbqt” format.

Moreover, the 3D structure of the synthesized chemical compounds was drawn using the Chemdraw tool and downloaded in a “.mol” format file. The best anticancer ligands were further energy minimized and converted into a “.pdbqt” format file. To validate the protocol, initially, the native ligands were redocked to the active sites of proteins, and after validation, all the synthesized small organic molecules were docked individually to the target proteins (3PE1, 4W6E, and 4HKI). Discovery studio visualizer was used to get the dimensions of the grid box (25 × 25 × 25 Å), and it was centered at their native ligand XYZ coordinates, that is, 22.77 × −29.95 × 14.46 Å for 3PE1; 15.54 × 20.84 × −27.09 for 4W6E; and −9.36 × −42.90 × 16.76 for 4HKI. The molecular docking was performed, and Kd (binding affinity) was analyzed as discussed previously [35,41]:

## 3. Results

### 3.1. Chemistry

#### Optimization and Synthesis of Compounds

The optimization of copper source and reductant was explored, and the findings are reported in Table 1. CuSO_4_·5H_2_O was found to be successful in producing Cu(I) in the presence of sodium ascorbate as a reductant and yielded the required product of **13aa′** (84% yield) among the Cu sources evaluated (Table 1, entry 2). However, Cu(OAc)_2_ was found to be the more effective and yielded the desired product 13aa′ in 96% yield (Table 1, entry 6). The major difference was observed in the rate of the reaction. In the presence of copper sulfate and sodium ascorbate/hydrazine hydrate, more time was consumed to complete the reaction as compared to copper acetate and hydrazine hydrate.

**Scheme 1:**
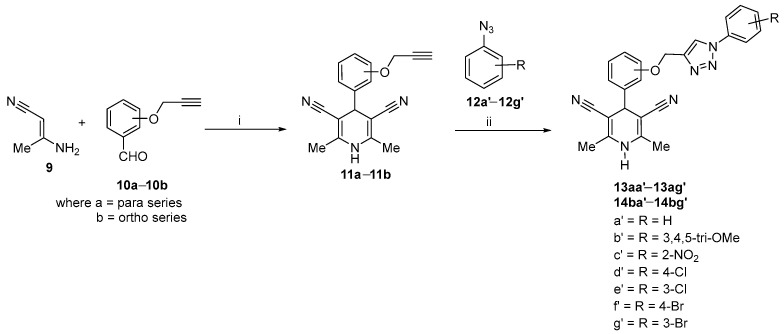
General route for synthesis of substituted 1,2,3-triazolyl-1,4-dihydropyridine derivatives (reagents and conditions: (i) gla. CH_3_COOH/N_2_ atm., reflux (ii) Cu(OAc)_2_/NH_2_NH_2_·H_2_O room temperature).

The synthesis of a 1,4-dihydropyridines-based substituted 1,2,3-triazoles **13aa′**–**13ag′** and **14ba′**–**14bg′** was achieved by following the synthetic route as shown in Scheme 1.

Having optimized the Cu sources and reductant (Table 1, entry 6), we explored the substrate scope for the CuAAC synthesis of 6-dimethyl-substituted-1*H*-1,2,3-triazol-4-yl)methoxy)phenyl)-1,4-dihydropyridine-3,5-dicarbonitrile 13 and 14. We obtained the excellent yield (84–96%) of final compounds in most of the cases without any requirement of further purification through chromatography (Table 2).

### 3.2. Biological Anticancer Activity

#### 3.2.1. In Vitro Cytotoxicity

**Figure 2:**
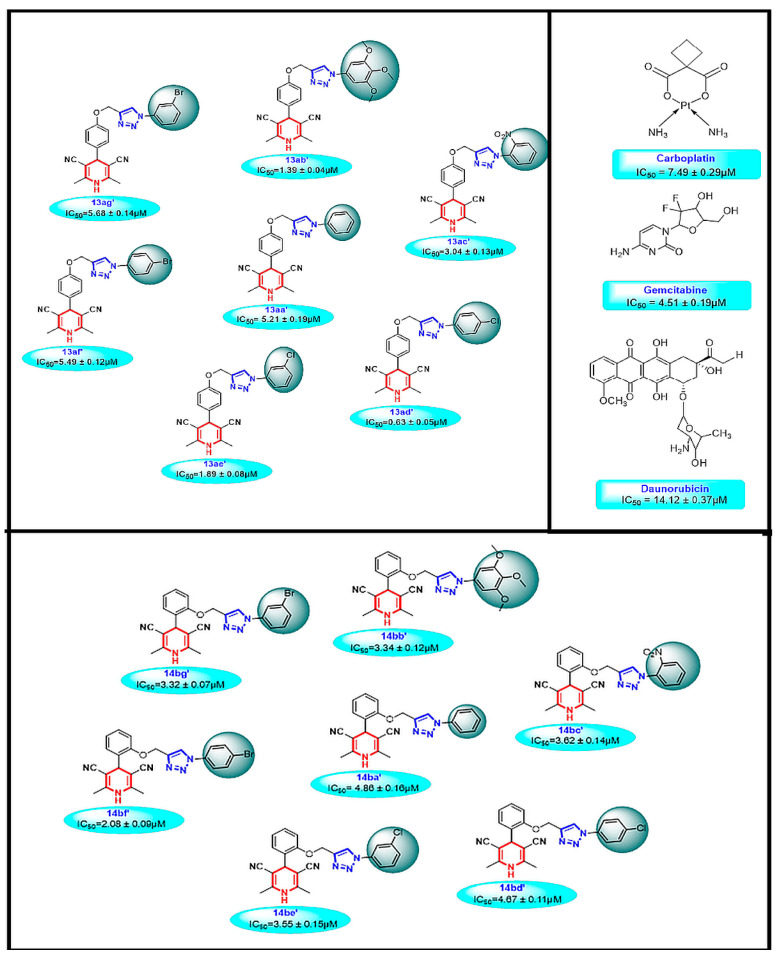
Cytotoxic potential of synthesized molecules and their structure activity relationship.

The cytotoxic efficacy of these synthesized compounds and reference drug carboplatin, gemcitabine, and daunorubicin in terms of IC_50_ is shown in Figure 2. From IC_50_ values, it is very much clear that most compounds have significant cytotoxicity in a dose-dependent manner (Figures S15 and S16; Table S1).

#### 3.2.2. Cell Cycle Inhibition

**Figure 3:**
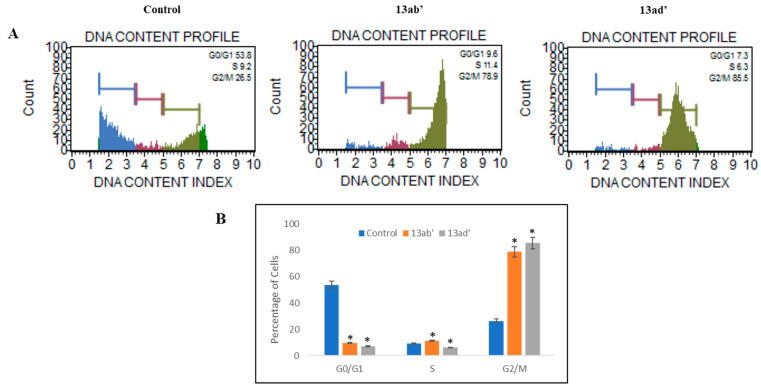
Analysis of subpopulation of Caco-2 cells in different cell cycle phases. (**A**) Effect of **13ab′** and **13ad′** on cell cycle represents those cells decrease in G0/G1 phase while increasing in G2/M phase. (**B**) Graphical representation of percentage cells in various phases (G0/G1, S, and G2/M) of cell cycle. All results were stated in mean ± SEM. * *p* < 0.05.

Results showed a significant increase in G2/M phases and decreased in the G0/G1 phase of the cell cycle when treated with these compounds for 24 h. All the results were statistically significant (*p* < 0.05) as compared to untreated cells (Figure 3), suggesting the regulating effect of these compounds on the cell cycle by arresting cell proliferation and progression in the G2/M phase resulting in the increased apoptosis in Caco-2 cells.

#### 3.2.3. Induction of Apoptosis

**Figure 4:**
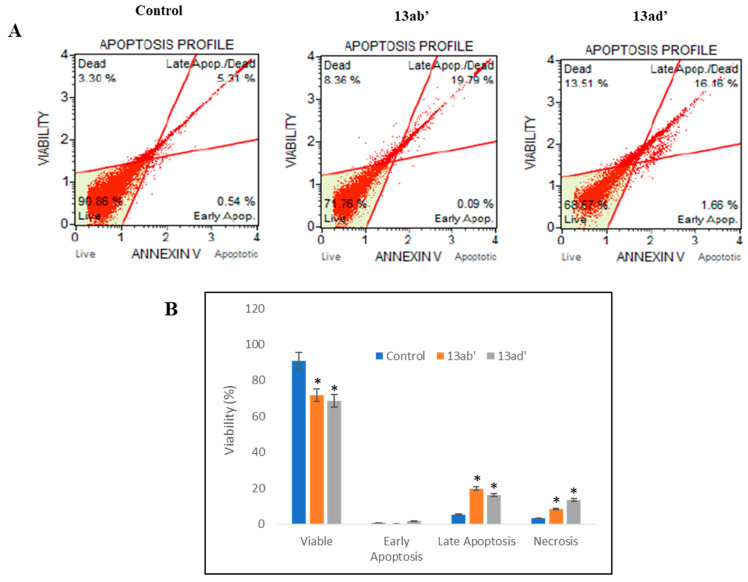
Apoptosis evaluation in Caco-2 cell line using Muse™ Annexin V and Dead Cell assay. (**A**) Represents apoptotic effect of **13ab′** and **13ad′** on Caco-2 cell line. Both compounds were displaying significant cell death necrosis along with the late apoptotic phase. Live cells with negative staining lie in the lower left. Early apoptotic cells with annexin V-positive/dead cell marker-negative lie in the lower right quadrant. Late apoptotic cells with annexin V-positive/dead cell marker-positive lie in the upper right, while dead cells with annexin V-negative/dead cell marker-positive lie in the upper left segment. (**B**) Graphical representation for the effect of **13ab′** and **13ad′** on viability of Caco-2 cell lines in different quadrant. *p*-value < 0.05 (*) shows significant results.

The results (Figure 4) revealed that control cells have a very low percentage of annexin V staining showing a higher percentage of viable cells. Both compounds exhibited a significant effect in decreasing the number of viable cells, while late apoptotic and necrotic cells were observed to be increased, which is suggestive of the fact that these compounds have the potential to induce apoptosis in Caco-2 cells. Nevertheless, in early apoptotic cells, no significant difference was observed.

### 3.3. Cheminformatics Molecular Interaction Analysis

The two best antiproliferation compounds (**13ab′** and **13ad′**) were docked in the active site pocket of target proteins (3PE1, 4W6E, and 4HKI) to get the binding energy (ΔG) and binding affinity (Kd), and the results are presented in Table 3.

**Figure 5:**
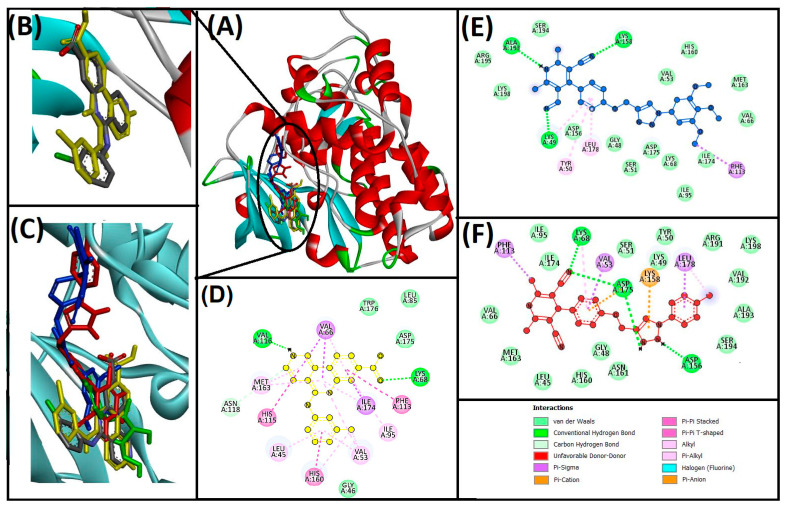
Interaction of target protein, protein kinase CK2α subunit (3PE1) with **13ab′** (blue), and **13ad′** (red). (**A**) Position of ligands and standard inhibitor in 3PE1, (**B**) superimpose image of redocked (yellow) and native ligand (gray) at specific native ligand site in 3PE1, (**C**) superimpose image of **13ab′** and **13ad′** including redocked native ligand in 3PE1, (**D**) interactions between 3PE1 and CX-4945 (redocked native ligand), (**E**) interactions between 3PE1 and **13ab′**, (**F**) interactions between 3PE1 and **13ad′**.

**Figure 6:**
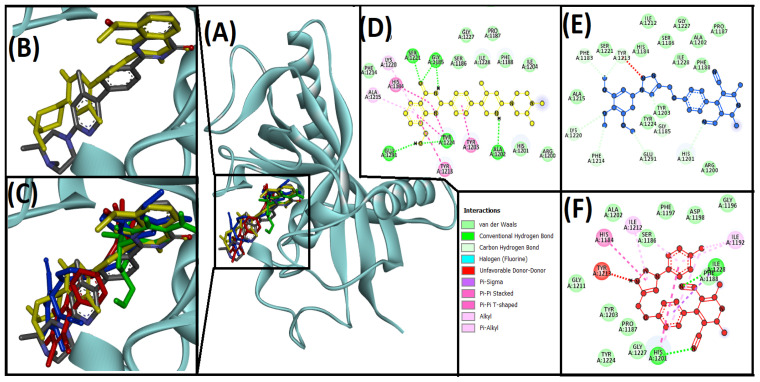
Interaction of target protein, tankyrase1 (4W6E) with, **13ab′** (blue), and **13ad′** (red). (**A**) Position of ligands and standard inhibitor in 4W6E, (**B**) superimpose image of redocked (yellow) and native ligand (gray) at specific native ligand site in 4W6E, (**C**) superimpose image of **13ab′**, and **13ad′** including redocked native ligand in 4W6E, (**D**) interactions between 4W6E and AZ6102 (redocked native ligand), (**E**) interactions between 4W6E and **13ab′**, (**F**) interactions between 4W6E and **13ad′**.

**Figure 7:**
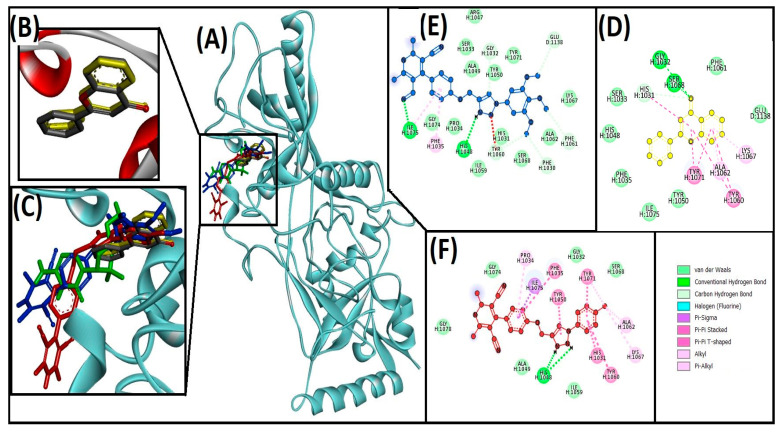
Interaction of target protein, tankyrase2 (4HKI) with **13ab′** (blue), and **13ad′** (red). (**A**) Position of ligands and standard inhibitor in 4HKI, (**B**) superimpose image of redocked (yellow) and native ligand (gray) at specific native ligand site in 4HKI, (**C**) superimpose image of **13ab′** and **13ad′** including redocked native ligand in 4HKI, (**D**) interactions between 4HKI and flavone (redocked native ligand), (**E**) interactions between 4HKI and **13ab′**, (**F**) interactions between 4HKI and **13ad′**.

Initially, the native ligands (CX-4945 for 3PE1, pyrrolopyrimidinone compound 25 (AZ6102) for 4W6E, and flavone for 4HKI) were redocked to the same site as in co-crystallized structure to validate the protocol, and our results illustrated that redocked ligands have nearly bound to the similar residues of the active site as in co-crystallized structure (Figure 5A–C, Figure 6A–C and Figure 7A–C, respectively). The synthesized compounds (**13ab′** and **13ad′**) were individually docked on the active site residues (Table 3 and Figure 5, Figure 6 and Figure 7). The 2D structure of redocked target proteins depicted through the DS visualizer revealed that the residues that participated in binding the CX-4945 (native ligand) in the active site of protein kinase CK2α with hydrogen bond and hydrophobic interactions are LEU45, VAL53, VAL66, LYS68, ILE95, PHE113, HIS115, VAL116, ASN118, HIS160, MET163, and ILE174 and exhibited the ΔG: −10.8 and Kd: 8.3 × 10^7^ M^−1^ for binding energy and binding affinity, respectively (Table 3 and Figure 5D).

Our molecular interaction results (Table 3) showed that the compound 13ab′ and 3PE1 complex was stabilized by four conventional hydrogen bond (donor-acceptor) between LYS49:HZ1–ligand:N, A:LYS158:HZ1–ligand:N, ligand:H–A:ALA193:O, and ligand:HN–ligand:O. In contrast, one carbon hydrogen nond interacted between ligand:C–ligand:N. Moreover, four hydrophobic interactions were formed to stabilize the complex between ligand:C–PHE113 (Pi-sigma), LYS49–ligand (alkyl), LEU178–ligand (alkyl), and TYR50–ligand (Pi-alkyl) (Figure 5E). The 13ad′ compound and 3PE1 complex was stabilized by four conventional hydrogen bond (donor-acceptor) between LYS68:HZ2–ligand:N, ASP175:HN–ligand:N, ligand:HN–ASP175:OD1, and ligand:HN–ASP156:OD2, and one Pi-cation; Pi-donor hydrogen bond between LYS158:HZ1–ligand. In contrast, one electrostatic (Pi-Anion) interaction was observed between ASP175:OD1–ligand. Moreover, five hydrophobic interactions were also observed between VAL53:CG2–ligand (Pi-sigma), LEU178:CD1–ligand (Pi-sigma), ligand:C–PHE113 (Pi-sigma), ligand:Cl–LEU178 (alkyl), and ligand–LYS68 (Pi-alkyl) (Figure 5F). It was found that one active site residue (PHE113) was involved in stabilizing the **13ab′** and 3PE1 complex, and three active site residues (VAL53, LYS68, and PHE113) were involved in stabilizing the **13ad′** and 3PE1 complex. The molecular docking also predicted the binding score of interacted molecules that revealed the binding energy and binding affinity of **13ad′** (−11 kcal/mol and 1.16 × 10^8^ M^−1^) is better than **13ab′** (−9.3 kcal/mol and 6.56 × 10^6^ M^−1^).

The inhibitors of tankyrases (tankyrase1 and tankyrase2) mainly interacted with nicotinamide subsite residues such as GLY1185 and SER1221 for tankyrase1 (GLY1032 AND SER1068 for tankyrase2) through hydrogen bond, TYR1224 in tankyrase1 (TYR1071 in tankyrase2) through Pi-Pi stacking, and PHE1188 in tankyrase1 (PHE1035 in tankyrase2). In contrast, some inhibitors interacted with adenosine subsite residues such as PHE1035, HIS1048, TYR1060, and TYR1071 in tankyrase2 [4]. The redocked native ligand (AZ6102) in the active site of human tankyrase1 (4W6E) interacted via hydrogen bond and hydrophobic interactions with residues HIS1184, GLY1185, ALA1202, TYR1203, TYR1213, ALA1215, LYS1220, SER1221, and TYR1224, which are similar residues that interacted with the native ligand in X-ray crystallographic structure suggested that results are reproducible and validated the docking protocol and exhibited the ΔG: −10.9, and Kd: 9.8 × 10^7^ M^−1^ for binding energy and binding affinity, respectively (Table 3 and Figure 6D). Our molecular interaction results demonstrate that the compound **13ab′** and 4W6E complex was stabilized by seven carbon hydrogen bond (donor-acceptor) between HIS1201:CA–ligand:N, LYS1220:CE–ligand:O, ligand:C–GLY1185:O, ligand:C–GLU1291:OE1, ligand:C–PHE1214:O, ligand:C–PHE1183:O, and ligand:C–TYR1213:O. Whereas one Unfavorable donor-donor interaction occurred between TYR1213:HN–ligand:N to stabilize the complex (Table 3 and Figure 6E). The 13ad′ compound and 4W6E complex were stabilized by two conventional hydrogen bond (donor-acceptor) between HIS1201:HD1–ligand:N, and ILE1228:HN–ligand:N. Several hydrophobic interactions were also involved stabilizing between the complex such as one Pi-sigma (ILE1228:CD1–ligand), one Pi-Pi stacked (HIS1201–ligand), one Pi-Pi T-shaped (HIS1184–ligand), one alkyl (ligand:Cl–ILE1192), and four Pi-alkyl (one between HIS1201–ligand:Cl, two between ligand–ILE1212, and one between ligand–ILE1192). Moreover, one unfavorable donor-donor interaction was also involved in stabilizing the complex between TYR1213:HN–ligand:HN (Table 3 and Figure 6F). It was found that three active site residues (GLY1185, TYR1213, and LYS1220) were involved in stabilizing the **13ab′** and 4W6E complex, and two active site residues (HIS1184 and TYR1213) were involved in stabilizing the **13ad′** and 4W6E complex. The molecular docking also predicted the binding score of interacted molecules that revealed the binding energy and binding affinity of **13ad′** (−10 kcal/mol and 2.14 × 10^7^ M^−1^) is better than **13ab′** (−8.6 kcal/mol and 2.01 × 10^6^ M^−1^).

Our results demonstrate that the complex of flavone (native ligand) and human tankyrase2 (4HKI) redocked in the active site was stabilized by the three hydrogen-bonded residues (GLY1032, SER1068, and HIS1031) and seven hydrophobic interactions with residues HIS1031, ALA1062, LYS1067, TYR1060, and TYR1071 of adenosine and nicotinamide subsites, which are in correspondence to the previous reports that validate the protocol [4]. Moreover, flavone exhibited the ΔG: −9.7 and Kd: 1.3 × 10^7^ M^−1^ for binding energy and binding affinity, respectively (Table 3 and Figure 7D). Our molecular interaction results demonstrate that the compound **13ab′** and 4HKI complex was stabilized by two conventional hydrogen bonds between ILE1075:HN–ligand:N, ligand:HN–HIS1048:O, and five carbon hydrogen bonds between ligand:C–GLY1032:O, ligand:C–GLU1138:OE1, ligand:C–PHE1061:O, ligand:C–PHE1030:O, and ligand:C–TYR1060:O. Moreover, this complex also stabilizes with two hydrophobic interactions between ILE1075–ligand (alkyl) and PHE1035–ligand (Pi-alkyl) (Table 3 and Figure 7E). The **13ad′** compound and 4HKI complex were stabilized by two conventional hydrogen bond (donor-acceptor) between ligand:HN–HIS1048:O. Several hydrophobic interactions were also involved in stabilizing the complex, such as one Pi-sigma between ILE1075:CD–ligand, four Pi-Pi stacked between HIS1031–ligand, TYR1060–ligand, TYR1071–ligand, and ligand–TYR1050, one Pi-Pi T-shaped between ligand–TYR1050, two alkyl between ALA1062–ligand:Cl, and ligand:Cl–LYS1067, and two Pi-alkyl between TYR1071–ligand:Cl, and ligand–PRO1034 (Table 3 and Figure 7F). It was found that two active site residues (GLY1032 and TYR1060) were involved in stabilizing the **13ab′** and 4W6E complex, and five active site residues (HIS1031, TYR1060, ALA1062, LYS1067, and TYR1071) were involved in stabilizing the **13ad′** and 4HKI complex. The molecular docking also predicted the binding score of interacted molecules with 4HKI protein, which revealed the binding energy and binding affinity of **13ad′** (−10.4 kcal/mol and 4.20 × 10^7^ M^−1^) is better than **13ab′** (−9 kcal/mol and 3.90 × 10^6^ M^−1^) (Table 3).

## 4. Discussion

The synthetic strategy was previously developed in our laboratory, in which various DHP’s linked with heterocyclic moieties were reported [24,25,26,42,43,44,45]. Keeping this observation in view, we aimed to improvise and prepare a library of dihydropyridines linked 1,2,3-triazoles via a simple, cost-effective, and expedient route in high yield, which was further evaluated for anticancer activity, and the mechanism of action was studied.

### 4.1. Chemistry

Because of dual action, the nature of Cu(I) salt is very essential for CuAAC reaction. In CuAAC, Cu(I) functions both as π and σ-electrophilic Lewis acid [46]. Several published reports show different copper sources were used individually or in combination with catalyst or ligand or via a green chemistry approach for the synthesis of triazole ring-based derivatives [30,47,48,49]. The CuI was found to be a better copper source in the presence of the ligand and DMF as solvent through the conventional method [49], while in the green chemistry approach, the CuSO_4_·5H_2_O and Cu(OAc)_2_ in the presence of reductants sodium ascorbate and hydrazine hydrate, respectively was earlier reported for better yield of click adduct in a short period of time [30,50,51]. From the previous literature, we hypothesized that hydrazine hydrate could be used as a reductant for CuSO_4_·5H_2_O or base for CuI and CuBr. Moreover, we also hypothesized that sodium ascorbate could be used as a reductant for Cu(OAc)_2_ because these combinations of copper sources and reductants might get a better yield in a minimum period of time via the green chemistry approach, and they were not reported earlier. We optimized the copper source (Cu(OAc)_2_) and reductant (hydrazine hydrate) for the synthesis of title compounds on the basis of higher yield within a short period of time. This reaction combination was earlier reported by Jiang et al. [30], but optimization of the various copper sources with different reductants was not reported. Therefore, in this study, several copper sources were investigated considering these facts, and the results are reported in Table 1. CuSO_4_·5H_2_O was found to be successful in producing Cu(I) in the presence of sodium ascorbate as a reductant and yielded the required product of **13aa′** (84% yield) among the Cu sources evaluated (Table 1, entry 2). However, Cu(OAc)_2_ was found to be the more effective and yielded desired product **13aa′** in 96% yield (Table 1, entry 6). The major difference was observed in the rate of the reaction. In the presence of copper sulfate and sodium ascorbate/hydrazine hydrate, more time was consumed to complete the reaction as compared to copper acetate and hydrazine hydrate. Hence, it was confirmed that hydrazine hydrate is working as a better reductant as compared to sodium ascorbate. In the absence of a reductant, the reaction did not progress, and traces were discovered at the end (Table 1, Entry 1, 4, 7, and 9). On the other hand, CuI and CuBr as different sources provide inferior yields in the existence of hydrazine hydrate.

The key 1,4-dihydropyridine component (11) was obtained by the condensation 2-aminocrotononitrile (9) with substituted propargylated benzaldehyde (10) in acetic acid by following the Hantzsch pyridine synthesis methodology [29]. The desired title compounds **13aa′**–**13ag′** and **14ba′**–**14bg′** were synthesized by the 1,3-diploar cycloaddition reaction between propargylated 1,4-dihydropyridines and substituted azides derivatives under click chemistry conditions in quantitative yields [30]. The existence of the propargyl group in 1,4-dihydropyridines (11) was confirmed by the presence of a signal at δ 5.20–5.26 for two protons of the –CH_2_ group. Similarly, the formation of the 1,2,3-triazole ring in the title compound was confirmed by the resonance of the proton in the triazolyl ring at a δ 8.70–9.00 (s, 1H, ArH) as a singlet.

Several reports suggested that the orthogonal analytical method (^1^H NMR) can be considered as a primary analytical tool for the detection of purity of the compounds in addition to structural identification in which the overlapping of the peaks or extra peaks suggests the presence of impurities in the compound [52,53]. In this study, the NMR spectra did not exhibit any extra peaks or any overlapping of the peaks in several compounds, but we observed a few compounds having extra peaks in very low intensity, and thus, we interpreted that the compounds are pure enough for the biological screening. We obtained the excellent yield (84–96%) of final compounds in most of the cases without any requirement of further purification through chromatography (Table 2).

### 4.2. Anticancer Activity

All compounds were screened against Caco-2 (colorectal adenocarcinoma) cell line to evaluate in vitro cytotoxicity. We noticed that all the title compounds except five compounds (**13aa′**, **13ag′**, **13af′**, **14ba′**, and **14bd′**) provided better cytotoxic activity than all the three reference drugs. Out of those compounds, **13ab′** (IC_50_ = 1.39 ± 0.04 µM) have second highest cytotoxic effect, which is around 5.37-fold, 3.22-fold, and 10.11-fold more active than carboplatin (IC_50_ = 7.49 ± 0.29 µM), gemcitabine (IC_50_ = 4.51 ± 0.19 µM) and daunorubicin (IC_50_ = 14.12 ± 0.37 µM). While **13ad′** provided the highest cytotoxic effect (IC_50_ = 0.63 ± 0.05 µM) is 11.75-fold, 7.06-fold, and 22.13-fold more active against the Caco-2 cell line than carboplatin, gemcitabine, and daunorubicin, respectively. Overall, all compounds have a significant (* *p* < 0.05) cytotoxic effect against the Caco-2 cell line and thus could be used as a promising target for developing anticancer drugs. Moreover, we noticed that the potency of **13ad′** is much better than the **13ab′**. From these compounds, we have used **13ab′** and **13ad′** further cellular assays. Conventional chemotherapeutic drugs are already known to modulate cancer cells growth by targeting cells in different phases of the cell cycle [54,55]. Thus, to investigate the underlying relationship of cytotoxicity of these compounds and cell cycle arrest, DNA accumulation at different phases of the cell cycle was analyzed using Muse^TM^ analyzer-based flow cytometric analysis. The significant increase in G2/M phases and decreased in G0/G1 phase of the cell cycle when treated with **13ab′** and **13ad′** compounds for 24 h, suggesting the regulating effect of these compounds on the cell cycle by arresting cell proliferation and progression in the G2/M phase resulting in the increased apoptosis in Caco-2 cells.

Furthermore, in apoptosis assay, we observed a significant decrease in the number of viable cells, while late apoptotic and necrotic cells were observed to be increased (Figure 4), which is suggestive of the fact that these compounds have the potential to induce apoptosis in Caco-2 cells. Several studies have reported the same cell cycle phase arrest in different cell lines [56,57]. Dhanasekhar et al. [58] described that lanatoside C has the potential to induce G2/M cell cycle arrest, which is leading toward suppressed cancer cell growth in three different cell lines, i.e., breast (MCF7), lung (A549), and liver (HepG2). They further described lanatoside C has the potential to arrest cells in the G2/M phase of the cell cycle by hindering the MAPK/Wnt/PAM signaling pathway, which is involved in its regulation. They also showed its capacity to induce apoptosis through targeting signaling pathways regulating DNA damage such as PI3K/AKT/mTOR. Another study revealed that dioscin inhibits the growth cancerous growth of osteosarcoma through G2/M cell cycle phase arrest and apoptosis by targeting the JNK/p38 pathway [59]. Thus, these compounds could be further used to check their efficiency to target different signaling pathways involved in cell cycle regulation as well as apoptosis for better insight into their mechanism of action.

### 4.3. Cheminformatics Molecular Interaction Analysis

For the computational molecular docking of ligand (**13ab′** and **13ad′**) with target proteins, the selection of PDB files (3PE1, 4W6E, and 4HKI) for the current study was due to their high resolution and their availability with their respective native ligand, which are the inhibitors and bound to the catalytic active site [6,60,61,62,63].

The molecular docking with protein kinase CK2α subunit (3PE1) predicted the binding score of interacted molecules that revealed the binding energy and binding affinity of **13ad′** (−11 kcal/mol and 1.16 × 10^8^ M^−1^) is better than **13ab′** (−9.3 kcal/mol and 6.56 × 10^6^ M^−1^). Our results are in correspondence with previous studies where it was reported that the residues of protein kinase CK2α subunit such as LYS68 and VAL116 formed hydrogen bonds, whereas VAL66, HIS160, and MET163 via hydrophobic bond interacted with CX-4945 [64,65]. Moreover, Oramas-Royo et al. docked some synthesized compounds with protein kinase CK2α and reported that the compounds fully occupied the active site in the adenine region (VAL53, VAL66, VAL116, and MET163), the hydrophobic region I (PHE113, ILE95, and ILE174), and hydrophobic region II (LEU45, and HIS115) [66]. Furthermore, the interaction of docked ligands and tankyrase1 (4W6E), as illustrated in Table 3 and Figure 6, revealed the binding energy and binding affinity of **13ad′** (−10 kcal/mol and 2.14 × 10^7^ M^−1^) is better than **13ab′** (−8.6 kcal/mol and 2.01 × 10^6^ M^−1^) are in correspondence with a previous study where it was reported that the 1,2,4-triazole derivative stabilized in adenosine and nicotinamide binding pocket of tankyrase1, which interacted with SER1186, PHE1188, ALA1191, ILE1192, LYS1195, HIS1201, MET1207, and TYR1213 [67]. Another study revealed that mainly GLU1291, SER1221, GLY1185, GLY1196, and ASP1198 residues were involved in stabilizing the tankyrase1 with natural compounds [63]. Our results demonstrate that the complex of flavone (native ligand) and human tankyrase2 (4HKI) redocked in the active site was stabilized by the three hydrogen-bonded residues (GLY1032, SER1068, and HIS1031) and seven hydrophobic interactions with residues HIS1031, ALA1062, LYS1067, TYR1060, and TYR1071 of adenosine and nicotinamide subsites, which are in correspondence to the previous reports that validate the protocol [4]. The molecular docking also predicted the binding score of interacted molecules with 4HKI protein, which revealed the binding energy and binding affinity of **13ad′** (−10.4 kcal/mol and 4.20 × 10^7^ M^−1^) is better than **13ab′** (−9 kcal/mol and 3.90 × 10^6^ M^−1^) (Table 3 and Figure 7).

Numerous proteins are involved in the progression of cancer where protein kinase CK2α and tankyrases (tankyrase1 and tankyrase2) were reported earlier as potential molecular targets to treat various human pathophysiological disorders and found to be overexpressed in several cancer cells [3,4]. The CK2α involved in tumorigenic, antiapoptotic, and failure of cell cycle arrest process by reducing the caspase activation potentiates the cancer drug-resistant controls unfolded protein response, reduces the tumor suppressor functions, and regulates the chaperone activity [3,5,68]. Whereas tankyrases (tankyrase1 and tankyrase2) are involved in various cellular pathways to promote cell proliferation, telomerase maintenance, helps in mitosis, and are involved in Wnt signaling. Recently, inhibition of tankyrases and/or protein kinase CK2α has emerged as an alluring approach for the finding of novel anticancer drugs [4,6,60,62,63,66,67]. Several inhibitors of CK2α were reported earlier, where CX-4945 exhibited the ATP-competitive mode of inhibition and showed anti-proliferative activity by promoting cell cycle arrest, inducing the caspase activity and apoptosis; hence, it was approved as an orphan drug by FDA for cancer treatment [60,62,66].

Therefore, the cheminformatics approach was applied in this study to understand the mechanism of action of antiproliferative and apoptotic properties of the best active compounds by inhibiting the target proteins involved in cell proliferation and antiapoptotic pathways. It is now evident that **13ad′** have better antiproliferation activity than the other compounds due to their better inhibition of target proteins involved in cancer proliferation.

## 5. Conclusions

In conclusion, using alkyne dihydropyridines and substituted azides, we have effectively established a novel, operationally easy, inexpensive, and environmentally friendly copper-catalyzed azide alkyne cyclization (CuAAC) reaction for the synthesis of functionalized triazoles. Among all the synthesized derivatives, **13ad′** was found to be the most favorable compound as it was showing 7.06-fold, 11.75-fold, and 22.13-fold better activity than gemcitabine, carboplatine, and daunorubicin against Caco-2 cell lines, respectively. Moreover, compound **13ad′** showed a significant effect on apoptosis in terms of cellular death and had the potential to arrest cells in G2/M phases of the cell cycle, resulting in increased apoptosis. The molecular docking study concluded that **13ad′** possess better binding affinity and interacted with the catalytic residues of target proteins involved in cell proliferation and antiapoptotic pathways. Nevertheless, more experiments may be needed to have a better idea about their mechanism of action against cancerous cells.

## Figures and Tables

**Table 1:** Optimization of Cu sources and reductants in CuAAC reaction *^a^*.

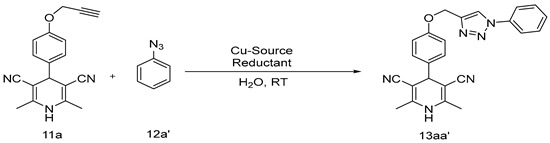				
Entry	Cu Sources	Reductants	Time (Hours)	% Yield *^b^*
1	CuSO_4_·5H_2_O	-	18	Traces
2	CuSO_4_·5H_2_O	Sodium Ascorbate	16	84
3	CuSO_4_·5H_2_O	Hydrazine Hydrate	3	69
4	Cu(OAc)_2_	-	24	Traces
5	Cu(OAc)_2_	Sodium Ascorbate	24	82
6	Cu(OAc)_2_	Hydrazine Hydrate	0.5	96
7	CuI	-	36	Traces
8	CuI	Hydrazine Hydrate	24	72
9	CuBr	-	22	Traces
10	CuBr	Hydrazine Hydrate	18	76

Reaction Conditions: *^a^* Starting materials 11a (1 mmol), **12a′** (1.1 mmol), 2 mmol of appropriate Cu sources, and 1 mmol reductants were stirred in 1 mL water in a sealed tube for the allocated time mentioned in the above table. *^b^* Isolated yields.

**Table 2:** Substrate scope for CuAAC reaction.

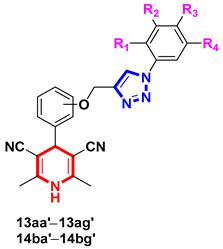							
Sr. No.	Compound	Position	R_1_	R_2_	R_3_	R_4_	% Yield
1	**13aa′**	para	H	H	H	H	96
2	**13ab′**	para	H	OCH_3_	OCH_3_	OCH_3_	87
3	**13ac′**	para	NO_2_	H	H	H	89
4	**13ad′**	para	H	H	Cl	H	84
5	**13ae′**	para	H	Cl	H	H	88
6	**13af′**	para	H	H	Br	H	92
7	**13ag′**	para	H	Br	H	H	89
8	**14ba′**	ortho	H	H	H	H	87
9	**14bb′**	ortho	H	OCH_3_	OCH_3_	OCH_3_	89
10	**14bc′**	ortho	NO_2_	H	H	H	85
11	**14bd′**	ortho	H	H	Cl	H	91
12	**14be′**	ortho	H	Cl	H	H	83
13	**14bf′**	ortho	H	H	Br	H	89
14	**14bg′**	ortho	H	Br	H	H	84

**Table 3:** Molecular docking scores and interactions of best hit compounds against 3PE1, 4HKI, and 4W6E.

3PE1					
Compounds	Hydrogen Bond	Hydrophobic	Unfavorable	ΔG (kcal/mol)	Kd (M^−1^)
**13ab′**	LYS49, LYS158, ALA193	PHE113, LYS49, LEU178, TYR50		−9.3	6.56 × 10^6^
**13ad′**	LYS68, ASP175, ASP156, LYS158	VAL53, LEU178, PHE113, LYS68		−11	1.16 × 10^8^
CX−4945 (native ligand)	LYS68, VAL116, ASN118	LEU45, VAL53, VAL66, ILE95, PHE113, HIS115, HIS160, MET163, ILE174		−10.8	8.3 × 10^7^
**4HKI**					
**Compounds**	**Hydrogen Bond**	**Hydrophobic**	**Unfavorable**	**ΔG (kcal/mol)**	**Kd (M^−1^)**
**13ab′**	ILE1075, HIS1048, GLY1032, GLU1138, PHE1061, PHE1030, TYR1060	ILE1075, PHE1035		−9	3.9 × 10^6^
**13ad′**	HIS1048	ILE1075, HIS1031, TYR1060, TYR1071, TYR1050, PHE1035, ALA1062, LYS1067, TYR1071, PRO1034		−10.4	4.20 × 10^7^
Flavone (native ligand)	GLY1032, SER1068, and HIS1031	HIS1031, ALA1062, LYS1067, TYR1060, TYR1071		−9.7	1.3 × 10^7^
**4W6E**					
**Compounds**	**Hydrogen Bond**	**Hydrophobic**	**Unfavorable**	**ΔG (kcal/mol)**	**Kd (M^−1^)**
**13ab′**	HIS1201, LYS1220, GLY1185, GLU1291, PHE1214, PHE1183, TYR1213			−8.6	2.01 × 10^6^
**13ad′**	HIS1201, ILE1228	ILE1228, HIS1201, HIS1184, ILE1192, HIS1201, ILE1212, ILE1198	TYR1213	−10	2.14 × 10^7^
AZ6102 (native ligand)	GLY1185, GLY1185, ALA1202, SER1221	HIS1184, TYR1203, TYR1213, ALA1215, LYS1220	TYR1224	−10.9	9.8 × 10^7^

## Data Availability

Not applicable.

## Supplementary Materials

The following supporting information can be downloaded at: https://www.mdpi.com/article/10.3390/life12040519/s1, Section S1. Synthetic procedure, Figures S1–S14. ^1^H and ^13^C NMR spectra of representative compounds. Figure S15: MTT assay graphs of percentage viability for para series compounds (**13aa′**–**13ag′**). Figure S16: MTT assay graphs of percentage viability for ortho series compounds (**14ba′**–**14bg′**). Table S1: Absorbance reading (570 nm) for MTT assay of **13ab′** and **13ad′** and their IC_50_ value.
