# Supplementary material for: Green Synthesis and Anticancer Potential of 1,4-Dihydropyridines-Based Triazole Derivatives: In Silico and In Vitro Study
Source: Life (Basel). 2022 Mar 31;12(4):519. doi: 10.3390/life12040519 (PMC9029820; doi:10.3390/life12040519)
Supplement: Supplementary file 1 [file life-12-00519-s001.zip › life-1608481-supplementary.pdf]

life

Article

Green Synthesis and Anticancer Potential of 1,4-Dihydropyridines-Based Triazole Derivatives: In Silico and In Vitro Study

Sabera Bijani [1,2], Danish Iqbal [3,5,*], Sheefa Mirza [4], Vicky Jain [1,2], Sadaf Jahan [3], Mohammed Alsaweed [3], Yahya Madkhali [3], Suliman A. Alsagaby [3], Saeed Banawas [3,5,6], Abdulrahman Algarni [7], Faris Alrumaihi [8], Rakesh M. Rawal [9], Wael Alturaiki [3] and Anamik Shah [2,10,*]

1 Department of Chemistry, Marwadi University, Rajkot 360005, Gujarat, India; sabera.bijani@marwadieducation.edu.in (S.B.); vicky.jain@marwadieducation.edu.in (V.J.)
2 Center of Excellence, National Facility for Drug Discovery Center, Department of Chemistry, Saurashtra University, Rajkot Gujarat-360005, India
3 Department of Medical Laboratory Sciences, College of Applied Medical Sciences, Majmaah University, Al Majmaah 11952, Saudi Arabia; s.jahan@mu.edu.sa (S.J.); m.alsaweed@mu.edu.sa (M.A.); y.madkhali@mu.edu.sa (Y.M.); s.alsaqaby@mu.edu.sa (S.A.A.); s.banawas@mu.edu.sa (S.B.); w.alturaiki@mu.edu.sa (W.A.)
4 Health and Basic Sciences Research Center, Majmaah University, Al Majmaah 15341, Saudi Arabia
5 Department of Internal Medicine, Faculty of Health Sciences, University of the Witwatersrand, Johannesburg 2193, South Africa; mirza.sheefa@yahoo.co.in
6 Department of Biomedical Sciences, Oregon State University, Corvallis, OR 97331, USA
7 Department of Medical Laboratory Technology, Faculty of Applied Medical Sciences, Northern Border University, Arar 91431, Saudi Arabia; abdulrahman.eid@nbu.edu.sa
8 Department of Medical Laboratories, College of Applied Medical Sciences, Qassim University, Buraydah 51425, Saudi Arabia; f_alrumaihi@qu.edu.sa
9 Department of Life Sciences, Gujarat University, Ahmedabad 380009, Gujarat, India; rakeshrawal@gujaratuniversity.ac.in
10 B/H Forensic Laboratory, Saurashtra University Karmachari Cooperative Society, Rajkot 360005, Gujarat, India
* Correspondence: da.mohammed@mu.edu.sa (D.I.); anamik_shah@hotmail.com (A.S.)

Citation: Bijani, S.; Iqbal, D.; Mirza, S.; Jain, V.; Jahan, S.; Alsaweed, M.; Madkhali, Y.; Alsagaby, S.A.; Banawas, S.; Algarni, A.; et al. Green Synthesis and Anticancer Potential of 1,4-Dihydropyridines-Based Triazole Derivatives: In Silico and In Vitro Study. *Life* **2022**, *12*, 519. https://doi.org/10.3390/life12040519

Academic Editor: Ju-Seop Kang

Received: 18 February 2022
Accepted: 21 March 2022
Published: 31 March 2022

Publisher's Note: MDPI stays neutral with regard to jurisdictional claims in published maps and institutional affiliations.

Copyright: © 2022 by the authors. Licensee MDPI, Basel, Switzerland. This article is an open access article distributed under the terms and conditions of the Creative Commons Attribution (CC BY) license (http://creativecommons.org/licenses/by/4.0/).

1. Synthetic procedure

1.1. General Information

All the starting materials, reagents, solvents, and catalysts were purchased from Aldrich or Merck or Loba Chem and were used without further purification. The chromatographic solvents used for isolation/purification of compounds were distilled prior to use. All chemicals were of analytical grade. For thin layer chromatography, 0.2-mm precoated plates of silica gel G60 F254 (Merck) were used. 1H (400 MHz) and 13C (100 MHz) NMR spectra were recorded on a BRUKER AVANCE II 400 MHz spectrometer using DMSO-d6 and CDCl3 as a solvent. The 1H and 13C chemical shifts are reported in δ ppm relative to tetramethylsilane using as internal reference. The following abbreviations were used to designate chemical shift multiplicities: s = singlet, b = broad, d = doublet, dd = double doublet, t = triplet, m = multiplet. The melting points were determined in open capillary tubes and are uncorrected.

1.2. Synthesis of 13aa′- 13ag′ and 14ba′ – 14bg′. The ^{1}H and ^{13}C NMR spectra of these compounds has been shown in Figure S1-S14.

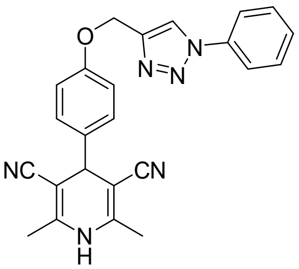

2,6-dimethyl-4-(4-((1-phenyl-1H-1,2,3-triazol-4-yl)methoxy)phenyl)-1,4-dihydropyridine-3,5-dicarbonitrile (13aa′).

Compound (13aa′) was prepared from 11a (300 mg, 1.0 mmol), 12a′ (135 mg, 1.1 mmol). White solid, Yield 360 mg, 85%, Melting point: 192 – 194 °C. 1H NMR (DMSO-d6) δ 9.51 (s, 1H, NH), 8.98 (s, 1H, ArH), 7.94 – 7.92 (m , 2H, ArH), 7.63 – 7.61 (m, 2H,ArH), 7.53 – 7.49 (m, 1H, ArH), 7.23 – 7.21. (m, 2H, ArH), 7.12 – 7.10 (m, 2H, ArH), 5.25 (s, 2H, CH2), (m, 4.36 (s, 1H, CH), 2.03 (s, 6H, 2×CH3). 13C NMR (DMSO-d6) δ 157.4, 148.4, 146.7, 146.3, 144.5, 144.3, 140.7, 137.1, 136.8, 131.4, 128.8, 126.1, 125.5, 123.2, 123.1, 120.6, 119.3, 114.8, 82.8, 60.9, 17.7.

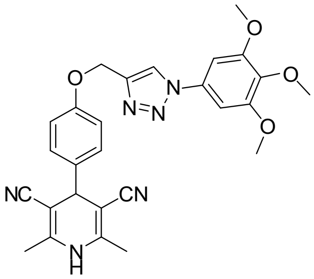

.

2,6-dimethyl-4-(4-((1-(3,4,5-trimethoxyphenyl)-1H-1,2,3-triazol-4-yl)methoxy)phenyl)-1,4-dihydropyridine-3,5-dicarbonitrile (13ab′).

Compound (**13ab′**) was prepared from **11a** (300 mg, 1.0 mmol), **12b′** (216 mg, 1.1 mmol). Off white solid, Yield 449 mg, 87 %, Melting point: 214 – 216 °C. ^{1}H NMR (DMSO-*d*$_6$) δ 9.51 (s, 1H, NH), 9.00 (s, 1H, ArH), 7.24 (s, 2H, ArH), 7.21 (d, J = 7.9 Hz, 2H, ArH), 7.11 (d, *J* = 7.9 Hz, 2H, ArH), 5.26 (s, 2H, CH_2), 4.36 (s, 1H, CH), 3.88 (s, 6H, 2×OCH_3), 3.71 (s, 3H, OCH_3), 2.03 (s, 6H, 2×CH_3). ^{13}C NMR (DMSO-*d*$_6$) δ 157.4, 153.4, 146.3, 143.7, 137.3, 136.8, 132.4, 128.8, 123.0, 119.3, 114.8, 98.0, 82.8, 61.1, 60.1, 56.2, 31.2, 17.7.

2,6-dimethyl-4-(4-((1-(2-nitrophenyl)-1H-1,2,3-triazol-4-yl)methoxy)phenyl)-1,4-dihydropyridine-3,5-dicarbonitrile (13ac′).

Compound (**13ac′**) was prepared from **11a** (300 mg, 1.0 mmol), **12c′** (170 mg, 1.0 mmol). Brown solid, Yield 418 mg, 89 %, Melting point: 202 – 204 °C. ^{1}H NMR (DMSO-*d*$_6$)

δ 9.52 (s, 1H, NH), 8.87 (s, 1H, ArH), 8.25- 8.23 (m, , 1H, ArH), 7.98 – 7.93 (m, 2H, ArH), 7.87 – 7.83 (m, 1H, ArH), 7.24 (d, *J* = 8.4 Hz, 2H, ArH), 7.13 (d, *J* = 8.4 Hz, 2H, ArH) 5.28 (s, 2H, CH_2), 4.37 (s, 1H, CH), 2.04 (s, 6H, 2×CH_3).^{13}C NMR (DMSO-d_6) δ157.5, 146.3, 144.0, 143.5, 136.8, 134.3, 131.2, 129.0, 128.8, 127.6, 125.9, 125.5, 119.3, 114.8, 82.9, 60.9, , 31.2, 17.7.

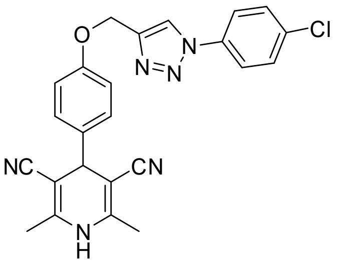

4-(4-((1-(4-chlorophenyl)-1H-1,2,3-triazol-4-yl)methoxy)phenyl)-2,6-dimethyl-1,4-dihydro-pyridine-3,5-dicarbonitrile (13ad′).

Compound (**13ad′**) was prepared from **11a** (300 mg, 1.0 mmol), **12d′** (175 mg, 1.1 mmol). Yellow solid, Yield 386 mg, 84 %, Melting point: 210 – 212 °C. ^{1}H NMR (DMSO-d_6) δ 9.50 (s, 1H, NH), 8.99 (s, 1H, ArH), 7.99 – 7.97 (m, 2H, ArH), 7.69 – 7.67 (m, 2H, ArH), 7.22 (d, *J* = 8.4Hz 2H, ArH), 7.12 (d, *J* = 8.4Hz, 2H, ArH), 5.26 (s, 2H, CH_2), 4.36 (s, 1H, CH), 2.04 (s, 6H, 2×CH_3).^{13}C NMR (DMSO-d_6) δ 157.4,146.3, 144.0,136.8,135.3, 133.0, 129.8, 128.8,122.8, 121.8, 119.3, 114.9, 82.9, 61.0,17.7.

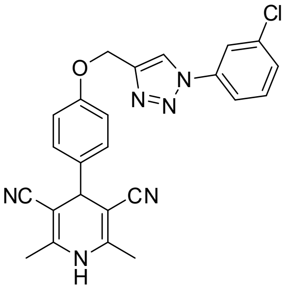

4-(4-((1-(3-chlorophenyl)-1H-1,2,3-triazol-4-yl)methoxy)phenyl)-2,6-dimethyl-1,4-dihydro-pyridine-3,5-dicarbonitrile (13ae′).

Compound (**13ae′**) was prepared from **11a** (300 mg, 1.0 mmol), **12e′** (175 mg, 1.2 mmol). Yellow solid, Yield 404 mg, 88 %, Melting point: 213 – 215 °C. ^{1}H NMR (DMSO-d_6) δ 9.44 (s, 1H, NH), 8.99 (s, 1H, ArH), 8.01 (s, 1H, ArH), 7.89 – 7.87 (m,1H, ArH), 7.59 – 7.57 (m,1H, ArH), 7.51 -7.49 (m, 1H, ArH), 7.15 (d, *J*=8.4Hz, 2H, ArH), 7.04 (d, *J*=8.4Hz, 2H, ArH), 5.19 (s, 2H, CH_2), 4.29 (s, 1H, CH), 1.96 (s, 6H, 2×CH_3). ^{13}C NMR (DMSO-d_6) δ 157.4, 146.7, 146.3, 144.5, 140.7, 136.8, 128.8, 125.5, 123.2, 120.6, 119.3, 114.8, 82.8, 60.9, 24.2, 17.7.

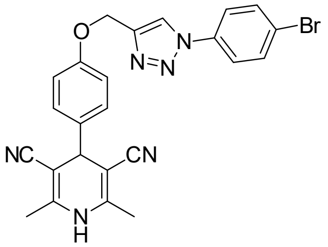

4-(4-((1-(4-bromophenyl)-1H-1,2,3-triazol-4-yl)methoxy)phenyl)-2,6-dimethyl-1,4-dihydro-pyridine-3,5-dicarbonitrile (13af′).

Compound (**13af′**) was prepared from **11a** (300 mg, 1.0 mmol), **12f′** (248 mg, 1.2 mmol). Brown solid, Yield 465 mg, 92 %, Melting point: 216 – 218 °C. ^{1}H NMR (DMSO-d_6) δ 9.25 (s, 1H, NH), 8.92 (s, 1H, ArH), 7.90 (d, J = 8.5 Hz, 2H, ArH), 7.82 (d, J = 8.5 Hz, 2H, ArH), 7.32 – 7.27 (m, 2H, ArH), 7.23 – 7.21 (m, 1H, ArH), 7.05 – 7.01 (m, 1H, ArH), 5.26 (s, 2H, CH_2), 4.76 (s, 1H, CH), 1.94 (s, 6H, 2×CH_3).^{13}C NMR (DMSO-d_6) δ 155.3, 146.9, 144.1, 135.8, 132.7, 131.8, 131.2, 129.7, 129.0, 122.5, 122.0, 121.4, 121.3, 119.4, 112.7, 81.6, 61.5, 17.6.

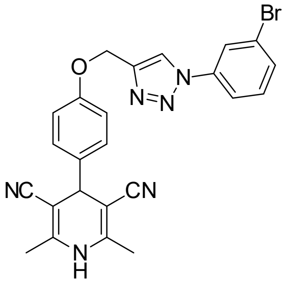

4-(4-((1-(3-bromophenyl)-1H-1,2,3-triazol-4-yl)methoxy)phenyl)-2,6-dimethyl-1,4-dihydropyridine-3,5-dicarbonitrile(13ag′).

Compound (**13ag′**) was prepared from **11a** (300 mg, 1.0 mmol), **12g′** (248 mg, 1.2 mmol). Brown solid, Yield 449 mg, 89 %, Melting point: 214 – 216 °C. ^{1}H NMR (DMSO-d_6) δ 9.51 (s, 1H, NH), 9.05 (s, 1H, ArH), 8.20 (s, 1H, ArH), 7.98 (d, *J*=7.8 Hz , 1H, ArH), 7.70 (d, *J*=7.8 Hz, 1H, ArH), 7.57 – 7.53 (m, 1H, ArH), 7.23 (d, *J*=8.4 Hz, 2H, ArH), 7.11 (d, *J*=8.4Hz, 2H, ArH), 5.26 (s, 2H, CH_2), 4.36 (s, 1H, CH), 2.03 (s, 6H, 2×CH_3). ^{13}C NMR (DMSO-d_6) δ 157.4, 148.4, 146.3, 144.0, 137.6, 136.8, 131.7, 131.4, 128.8, 122.9, 122.6, 122.4, 119.3, 119.0, 114.8, 82.8, 61.0, 17.7.

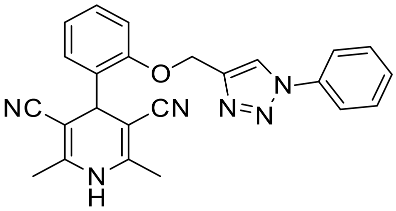

2,6-dimethyl-4-(2-((1-phenyl-1H-1,2,3-triazol-4-yl)methoxy)phenyl)-1,4-dihydropyridine-3,5-dicarbonitrile (14ba′).

Compound (**14ba′**) was prepared from **11b** (300 mg, 1.0 mmol), **12a′** (135 mg, 1.1 mmol). Cream solid, Yield 368 mg, 87 %, Melting point: 162 – 164 °C. ^{1}H NMR (DMSO-d_6) δ 9.47 (s, 1H, NH), 8.88 (s, 1H, ArH), 7.92 – 7.91 (m, 2H, ArH), 7.61 – 7.59 (m, 2H, ArH), 7.37 – 7.34 (m, 1H, ArH), 7.32 – 7.29 (m, 2H, ArH), 7.05 – 7.03 (m, 2H, ArH), 5.24 (s, 2H, CH_2), 4.77 (s, 1H, CH), 1.95 (s, 6H, 2×CH_3). ^{13}C NMR (DMSO-d_6) δ155.3, 147.0, 143.9, 136.6, 132.1, 131.8, 129.8, 129.7, 129.6, 129.4, 129.0, 128.8, 128.6, 128.2, 123.5, 122.5, 121.6, 121.3, 120.1, 119.4, 119.2, 81.6, 78.1, 61.6, 17.7.

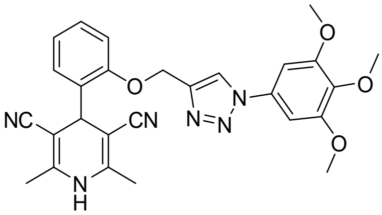

2,6-dimethyl-4-(2-((1-(3,4,5-trimethoxyphenyl)-1H-1,2,3-triazol-4-yl)methoxy)phenyl)-1,4-dihydropyridine-3,5-dicarbonitrile(14bb′).

Compound (**14bb′**) was prepared from **11b** (300 mg, 1.0 mmol), **12b′** (216 mg, 1.1 mmol). Brown solid, Yield 460 mg, 89 %, Melting point: 180 – 182 °C. ^{1}H NMR (DMSO-*d6*) δ 9.25 (s, 1H, NH), 8.86 (s, 1H, ArH), 7.30 – 7.24 (m, 3H, ArH), 7.19 (s, 2H, ArH), 7.02 – 7.01 (m, 1H, ArH), 5.26 (s, 2H, CH_2), 4.73 (s, 1H, ArH), 3.85 (s, 6H, 2×OCH_3), 3.80 (s, 3H, OCH_3), 1.94 (s, 6H, 2×CH_3). ^{13}C NMR (DMSO-*d6*) δ 155.2, 153.4, 147.0, 143.7, 137.3, 132.4, 131.9, 129.7, 129.0, 122.8, 121.4, 119.4, 112.7, 98.1, 81.6, 61.6, 60.1, 56.2, 31.2, 17.6.

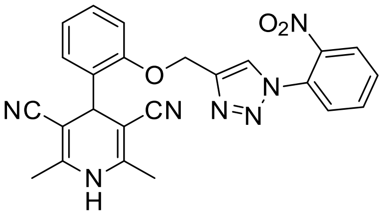

2,6-dimethyl-4-(2-((1-(2-nitrophenyl)-1H-1,2,3-triazol-4-yl)methoxy)phenyl)-1,4-dihydro-pyridine-3,5-dicarbonitrile(14bc′).

Compound (**14bc′**) was prepared from **11b** (300 mg, 1.0 mmol), **12c′** (170 mg, 1.0 mmol). Brown solid, Yield 399 mg, 85 %, Melting point: 170 – 172 °C. ^{1}H NMR (DMSO-*d6*) δ 9.28 (s, 1H, NH), 8.80 (s, 1H, ArH), 8.25 – 8.23 (m, 1H, ArH), 8.00 – 7.96 (m, 1H, ArH), 7.90 – 7.84 (m, 2H, ArH), 7.34 – 7.29 (m, 2H, ArH), 7.25 – 7.23 (m, 1H, ArH), 7.07 – 7.03 (m, 1H, ArH), 5.29 (s, 1H, CH_2), 4.78 (s, 1H, ArH), 1.97 (s, 6H, 2XCH_3). ^{13}C NMR (DMSO-*d6*) δ 155.2, 147.0, 144.0, 143.7, 134.3, 131.8, 131.1, 129.7, 129.0, 127.4, 125.5, 121.5, 119.4, 112.8, 81.7, 61.6, 17.6.

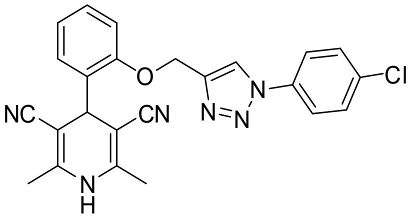

4-(2-((1-(4-chlorophenyl)-1H-1,2,3-triazol-4-yl)methoxy)phenyl)-2,6-dimethyl-1,4-dihydro-pyridine-3,5-dicarbonitrile (14bd′).

Compound (**14bd′**) was prepared from **11b** (300 mg, 1.0 mmol), **12d′** (175 mg, 1.0 mmol). Yellow solid, Yield 418 mg, 91%, Melting point: 175- – 177 °C. ^{1}H NMR (DMSO-*d6*) δ 9.25 (s, 1H, NH), 8.92 (s, 1H, ArH), 7.96 (d, *J*=8.4 Hz, 2H, ArH), 7.69 (d, *J*=8.4 Hz, 2H, ArH), 7.32-7.27 (m, 2H, ArH), 7.23 – 7.21 (m,1H, ArH), 7.05 – 7.01 (m, 1H, ArH), 5.26 (s, 2H, CH_2), 4.70 (s, 1H, CH), 1.94 (s, 6H, 2 X CH_3), ^{13}C NMR (DMSO-*d6*) δ 155.3, 154.5, 147.0, 146.9, 144.1, 135.4, 132.9, 132.1, 131.8, 129.8, 129.0, 128.3, 122.5, 121.7, 119.4, 112.8, 112.7, 81.7, 61.5, 56.0, 17.6.

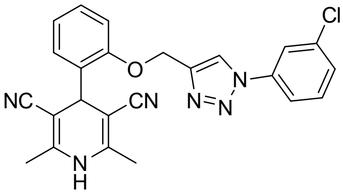

4-(2-((1-(3-chlorophenyl)-1H-1,2,3-triazol-4-yl)methoxy)phenyl)-2,6-dimethyl-1,4-dihydro-pyridine-3,5-dicarbonitrile (14be′).

Compound (**14be′**) was prepared from **11b** (300 mg, 1.0 mmol), **12e′** (175 mg, 1.0 mmol). Yellow solid, Yield 381 mg, 83 %, Melting point: 178 – 180 °C. ^{1}H NMR (DMSO-*d6*)δ 9.24 (s, 1H, NH), 8.97 (s, 1H, ArH), 8.06 (s, 1H, ArH), 7.99 – 7.93 (m, 1H, ArH), 7.66 –

7.62 (m, 1H, ArH), 7.58 – 7.56 (m, 1H, ArH), 7.43-7.28 (m, 2H, ArH), 7.24 – 7.22 (m, 1H, ArH), 7.06 – 7.02 (m, 1H, ArH), 5.27 (s, 2H, CH_2), 4.77 (s, 1H, CH), 1.95 (s, 6H, 2×CH_3). ^{13}C NMR (DMSO-d_6) δ 155.3, 146.9, 144.1, 137.6, 134.1, 131.8, 131.5, 129.7, 129.0, 128.4, 122.6, 121.3, 119.9, 119.4, 118.6, 112.7, 81.6, 61.5, 17.6.

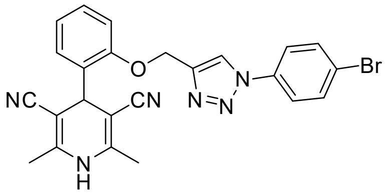

4-(2-((1-(4-bromophenyl)-1H-1,2,3-triazol-4-yl)methoxy)phenyl)-2,6-dimethyl-1,4-dihydro-pyridine-3,5-dicarbonitrile (14bf′).

Compound (**14bf′**) was prepared from **11b** (300 mg, 1.0 mmol), **12f′** (200 mg, 1.1 mmol). Brown solid, Yield 449 mg, 89 %, Melting point: 172 – 174 °C. ^1H NMR (DMSO-d_6) δ 9.25 (s, 1H, NH), 8.92 (s, 1H, ArH), 7.88 (d, *J* = 8.5 Hz, 2H, ArH), 7.82 (d, *J* = 8.5 Hz, 2H, ArH), 7.32 – 7.27 (m, 2H, ArH), 7.23 – 7.21 (m, 1H, ArH), 7.05 – 7.01 (m, 1H, ArH) 5.26 (s, 2H, CH_2), 4.76 (s, 1H, ArH), 1.94 (s, 6H, 2×CH_3). ^{13}C NMR (DMSO-d_6) δ 155.3, 146.9, 144.1, 135.8 132.7, 131.8, 131.2, 129.7, 129.0, 122.5, 122.0, 121.4, 121.3, 119.4, 112.7, 81.6, 61.5, 17.5.

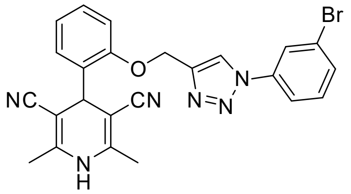

4-(2-((1-(3-bromophenyl)-1H-1,2,3-triazol-4-yl)methoxy)phenyl)-2,6-dimethyl-1,4-dihydro-pyridine-3,5-dicarbonitrile (14bg′).

Compound (**14bg′**) was prepared from **11b** (300 mg, 1.0 mmol), **12g′** (200 mg, 1.1 mmol). Brown solid, Yield 424 mg, 84 %, Melting point: 177 – 179 °C. ^1H NMR (DMSO-d_6) δ 9.25 (s, 1H, NH), 9.00 (s, 1H, ArH), 8.20 (s, 1H, ArH), 8.07 - 7.93 (m, 1H, ArH), 7.70 – 7.58 (m, 2H, ArH), 7.40 – 7.25 (m, 3H, ArH), 7.10 -7.05 (m,1H, ArH), 5.30 (s, 2H, CH_2), 4.79 (s, 1H, CH) 1.96 (s, 6H, 2×CH_3). ^{13}C NMR (DMSO-d_6) δ155.3, 146.9, 144.1, 137.7, 131.8, 131.7, 131.3, 129.7, 129.0, 122.6, 122.4, 121.3, 119.4, 119.0, 112.7, 81.6, 61.5, 17.5.

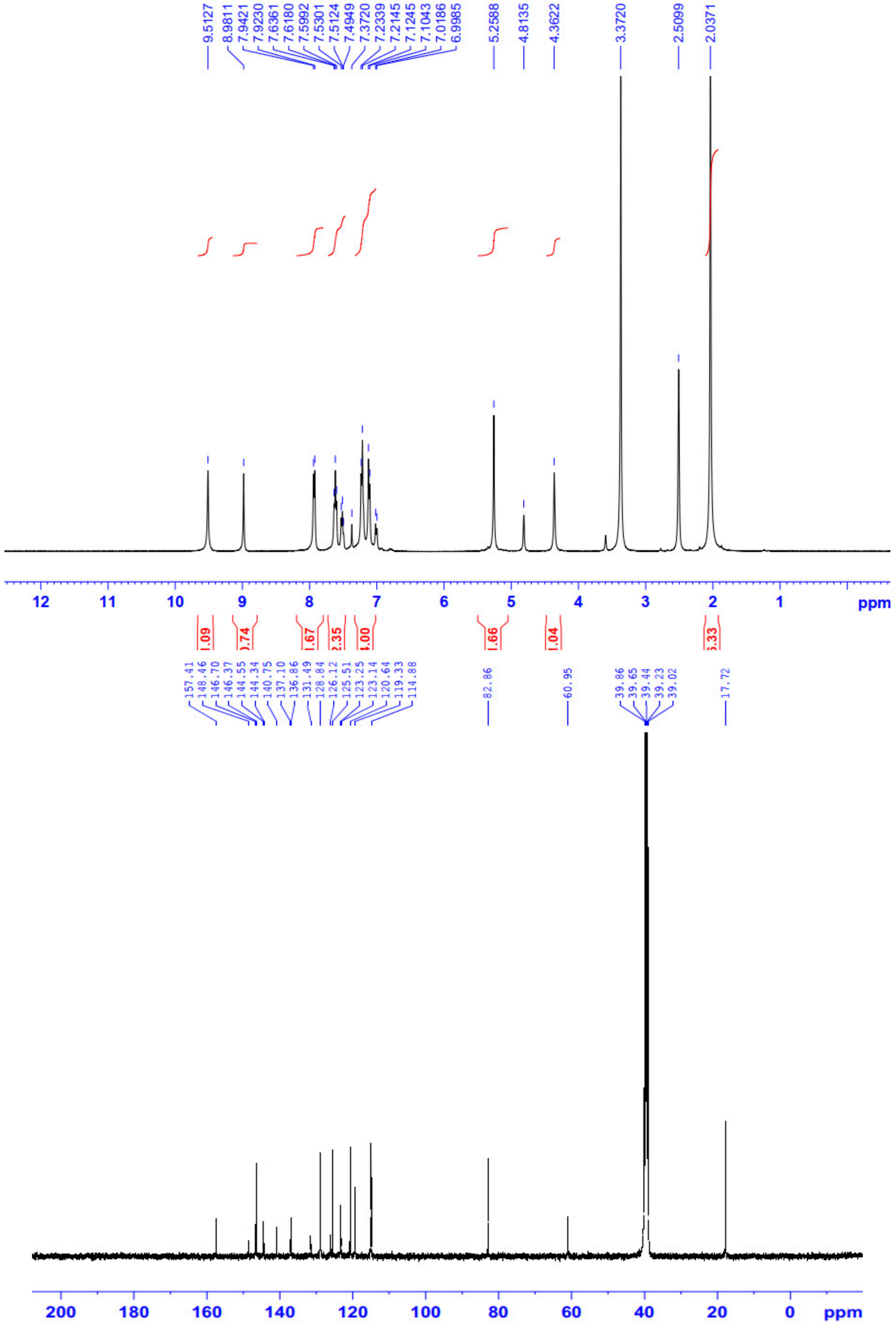

Figure S1. ^{1}H and ^{13}C NMR spectrum of 13aa′ (400 MHz, DMSO-*d*$_6$).

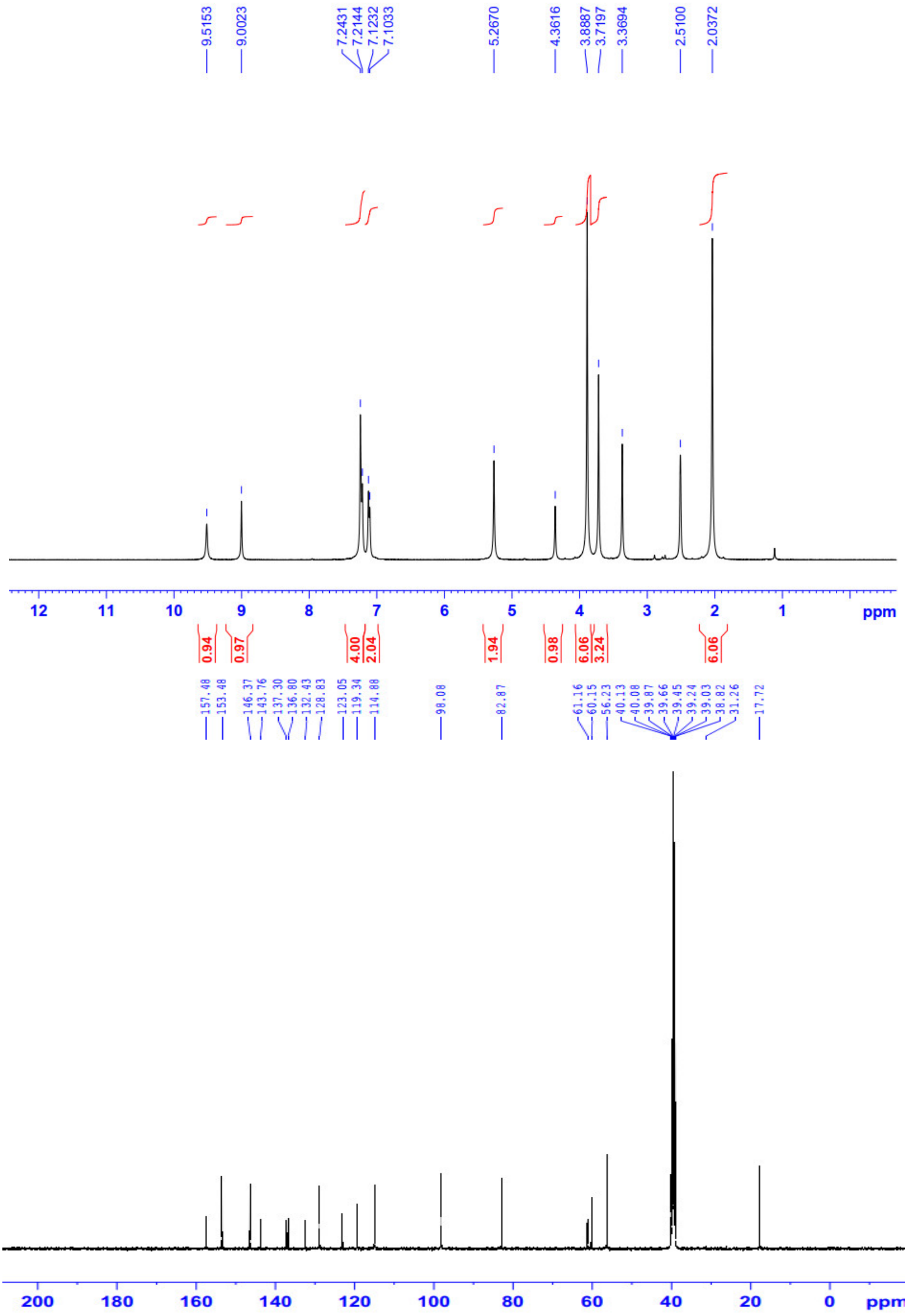

Figure S2. ^{1}H and ^{13}C NMR spectrum of 13ab′ (400 MHz, DMSO-d_6).

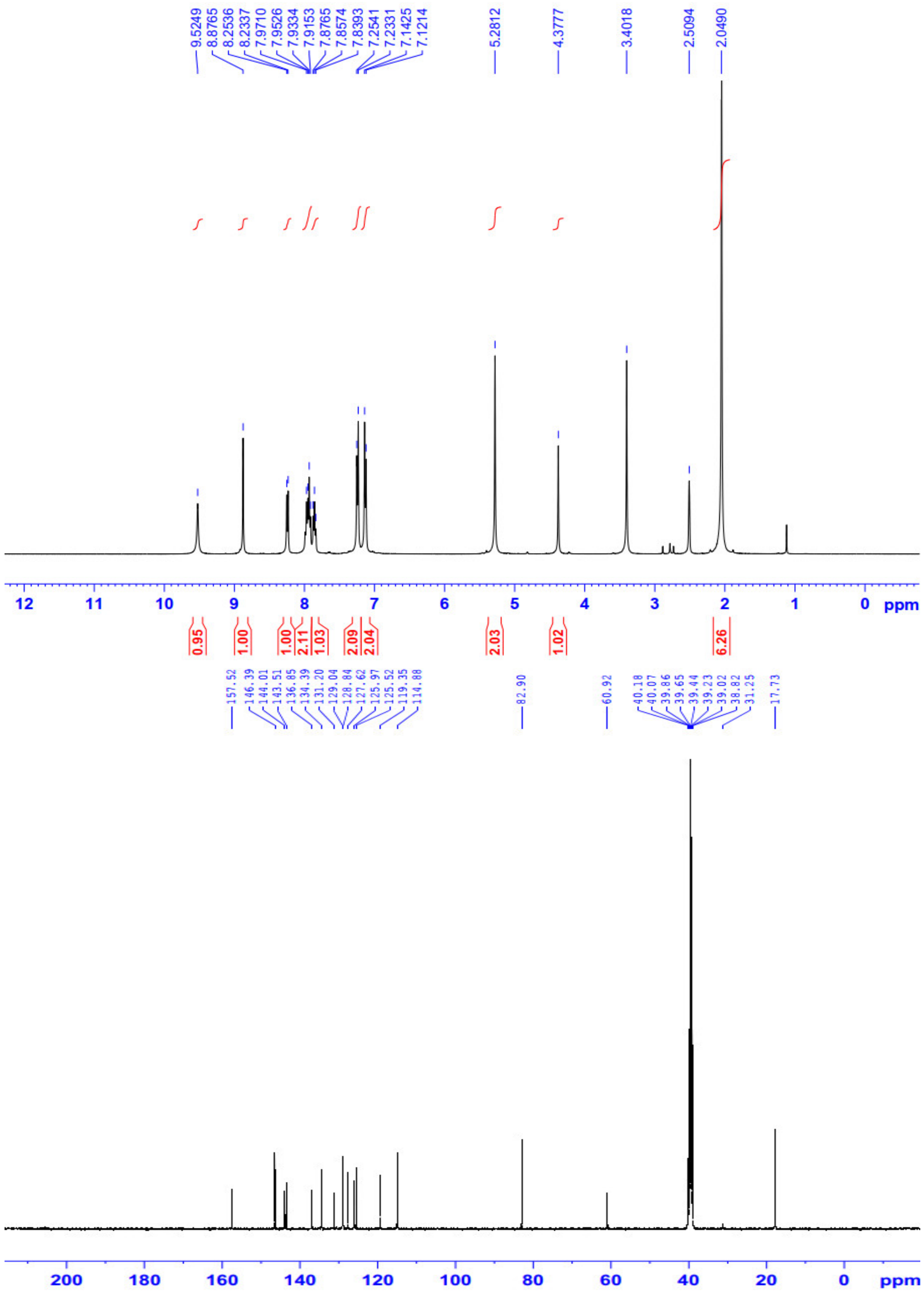

Figure S3. ^{1}H and ^{13}C NMR spectrum of 13ac′ (400 MHz, DMSO-d_6).

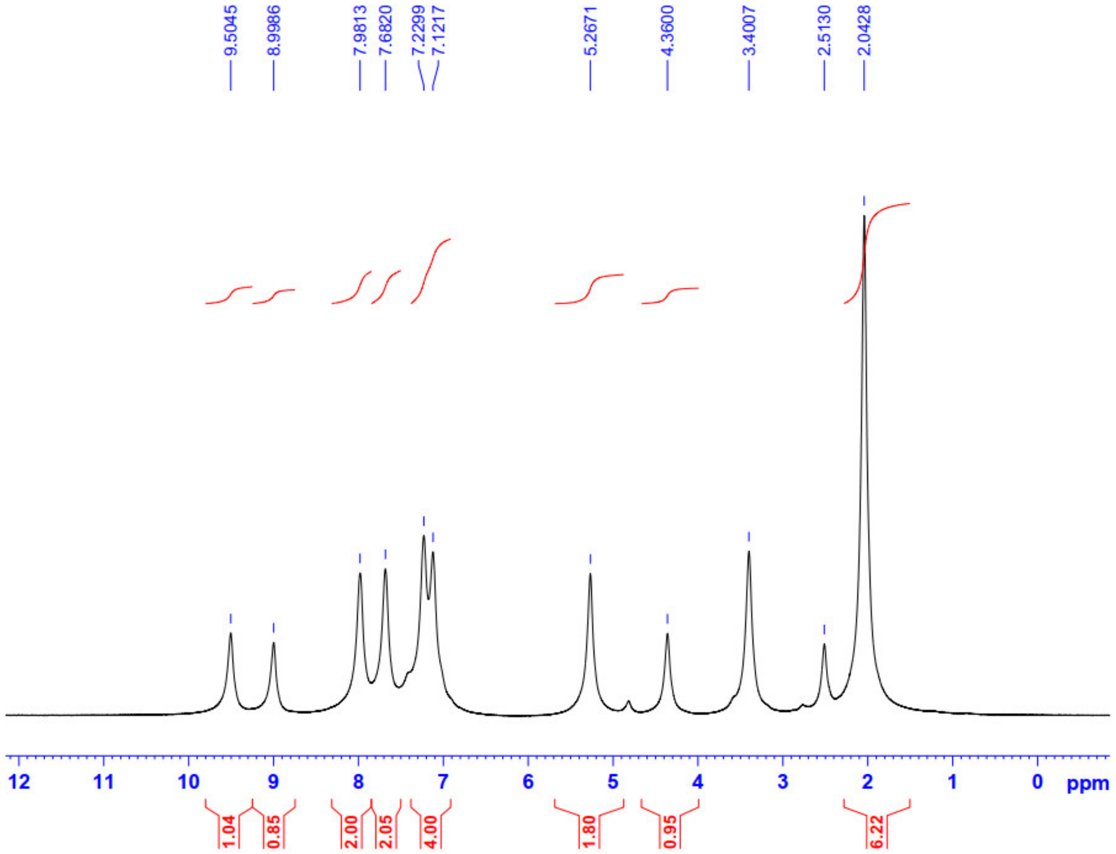

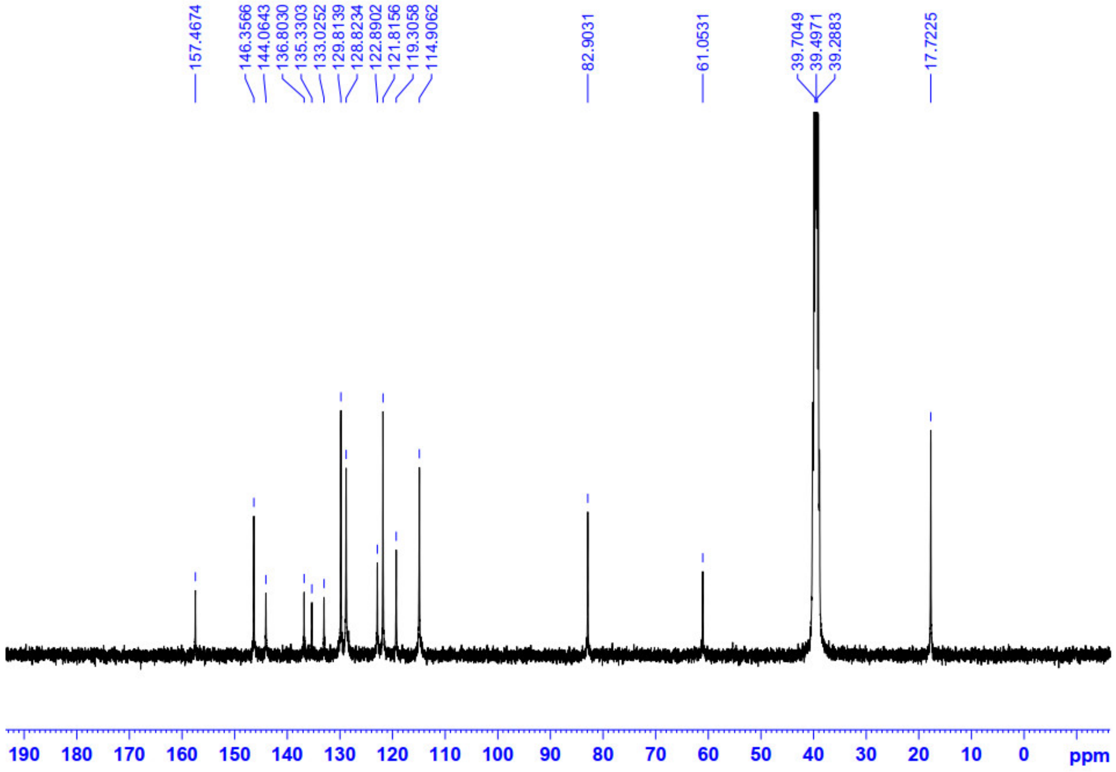

Figure S4. ^{1}H and ^{13}C NMR spectrum of 13ad′ (400 MHz, DMSO-d_6).

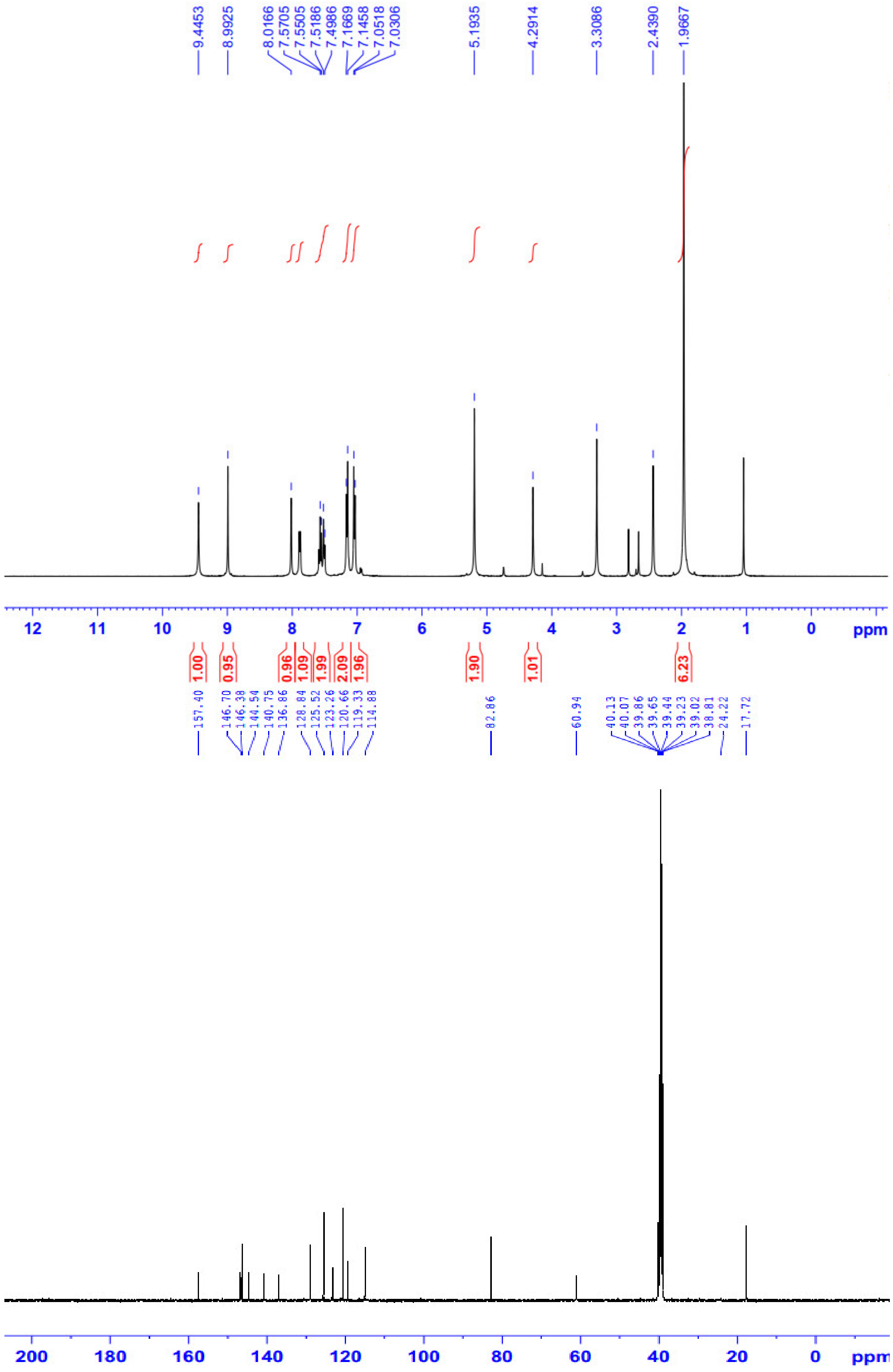

Figure S5. ^1H and ^{13}C NMR spectrum of 13ae′ (400 MHz, DMSO-d_6).

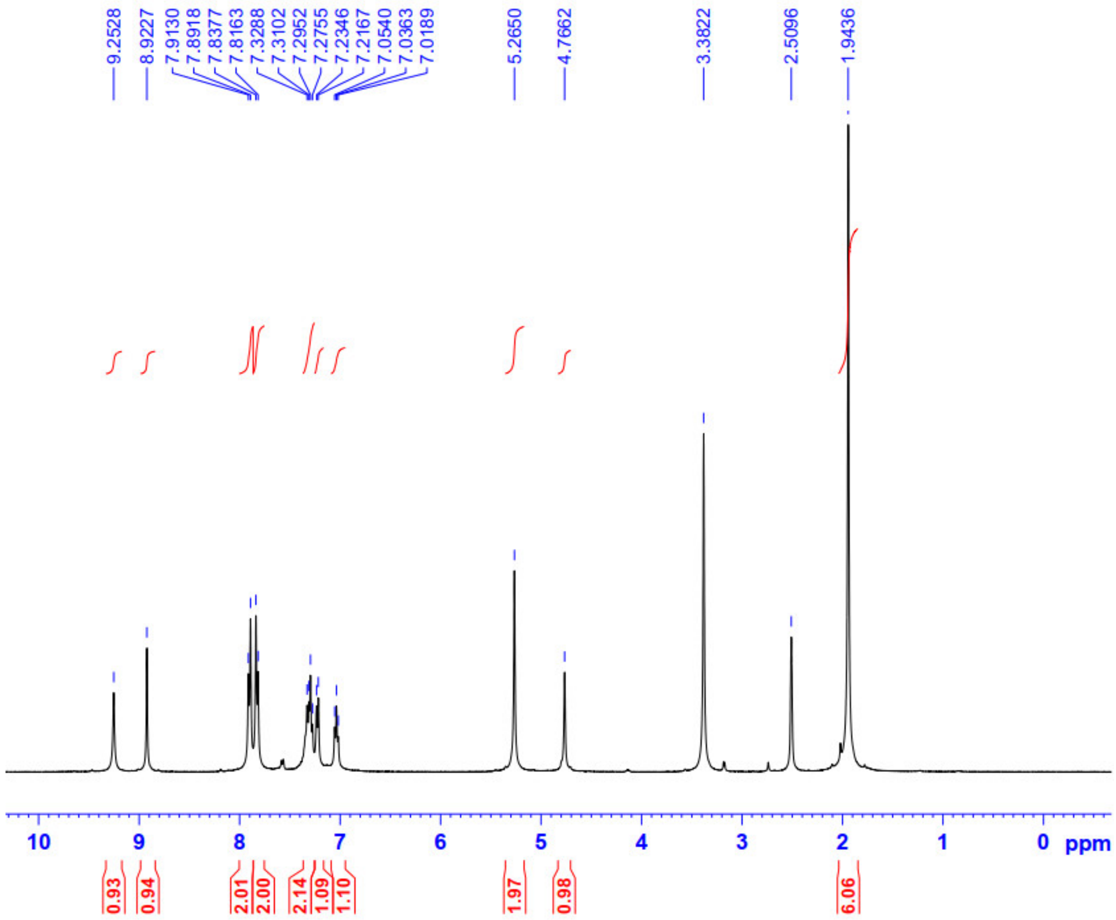

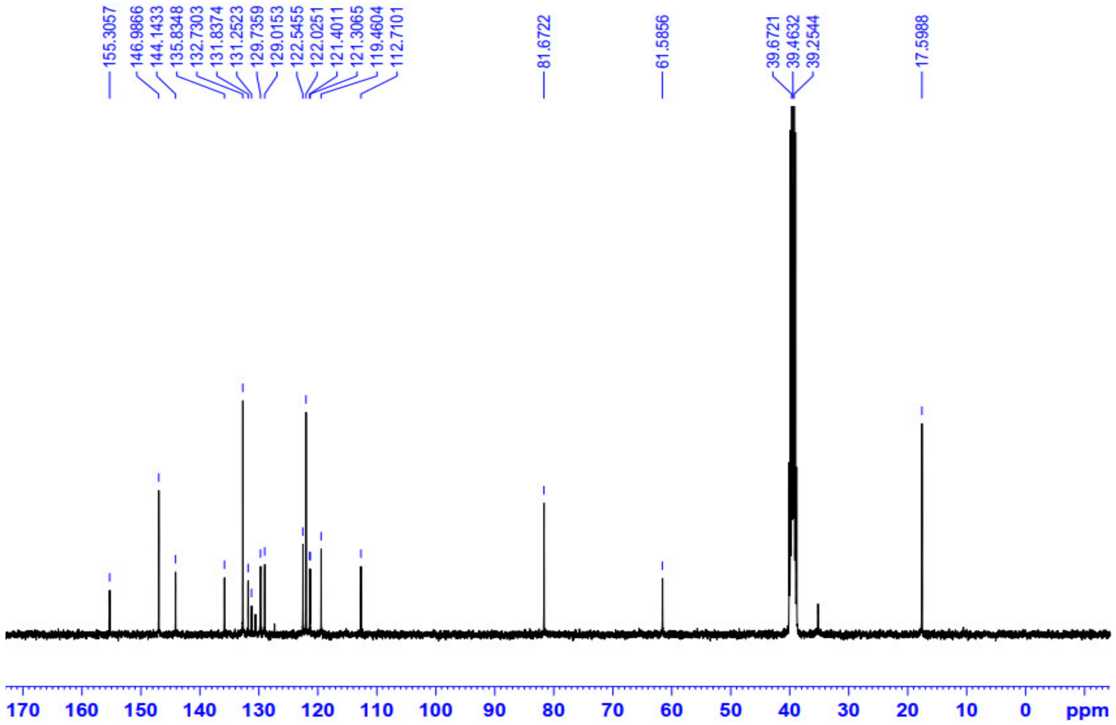

Figure S6. ^{1}H and ^{13}C NMR spectrum of 13af′ (400 MHz, DMSO-d_6).

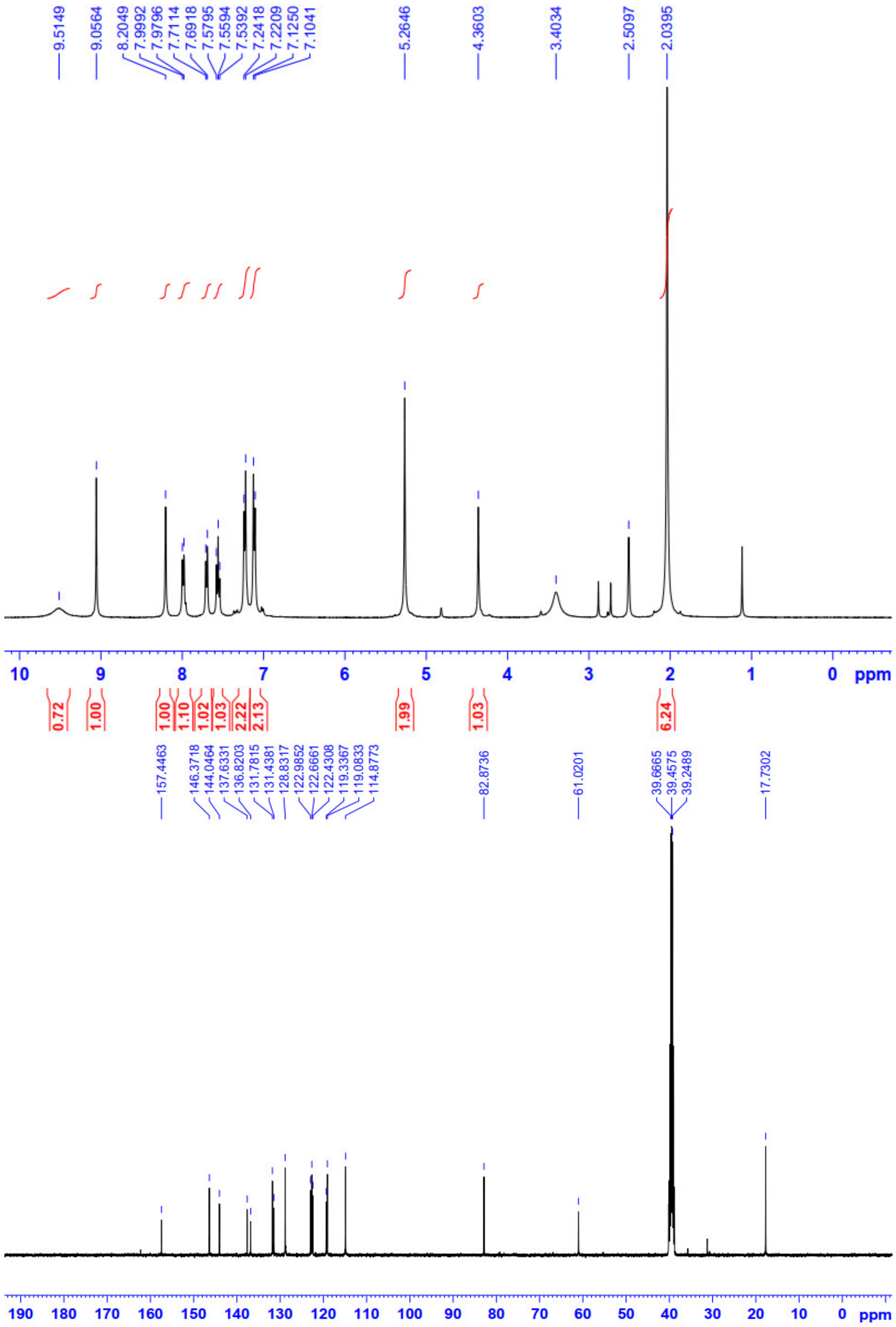

Figure S7. ^{1}H and ^{13}C NMR spectrum of 13ag′ (400 MHz, DMSO-d_6).

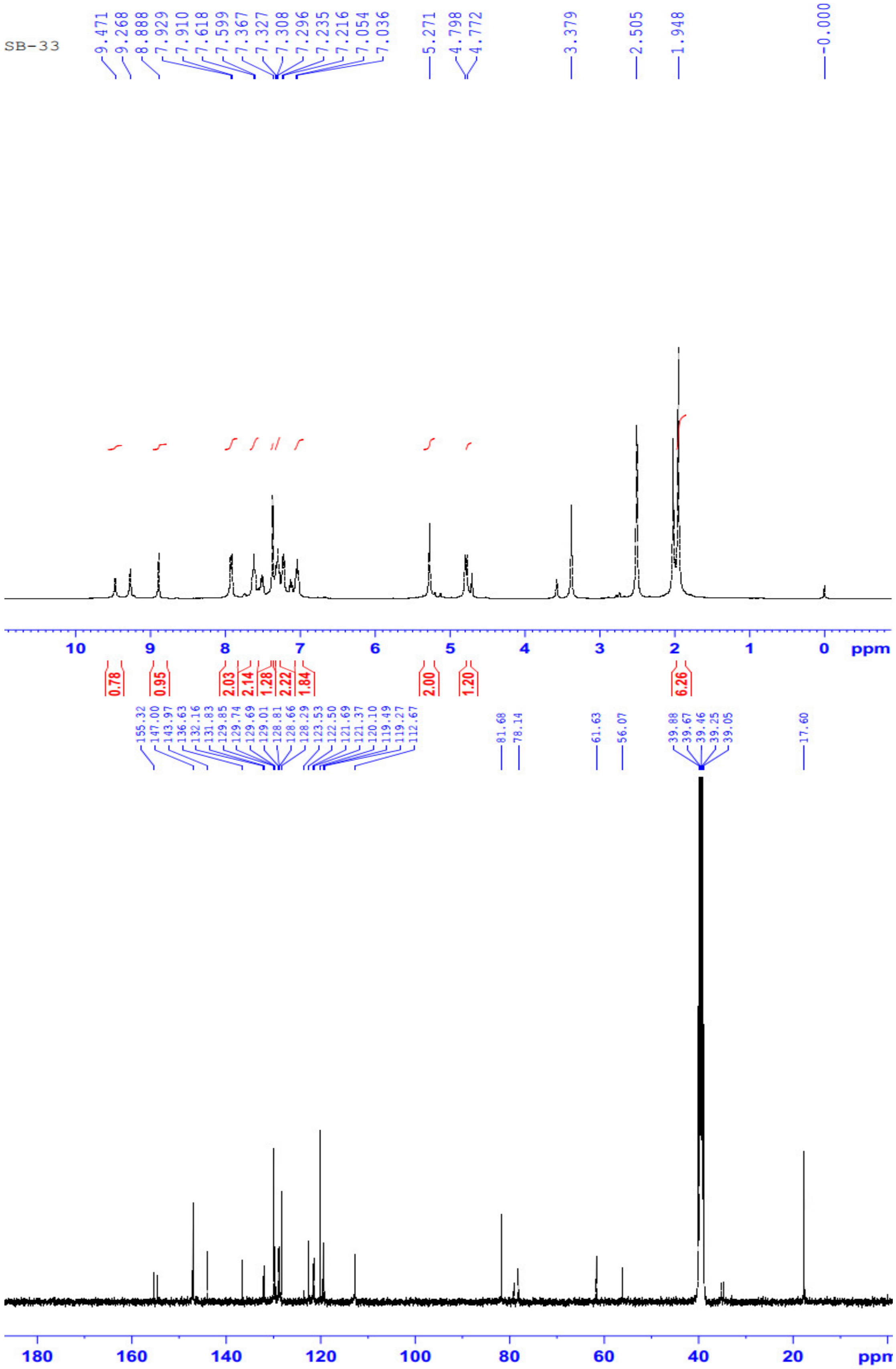

Figure S8. ^{1}H and ^{13}C NMR spectrum of 14ba′ (400 MHz, DMSO-d_6).

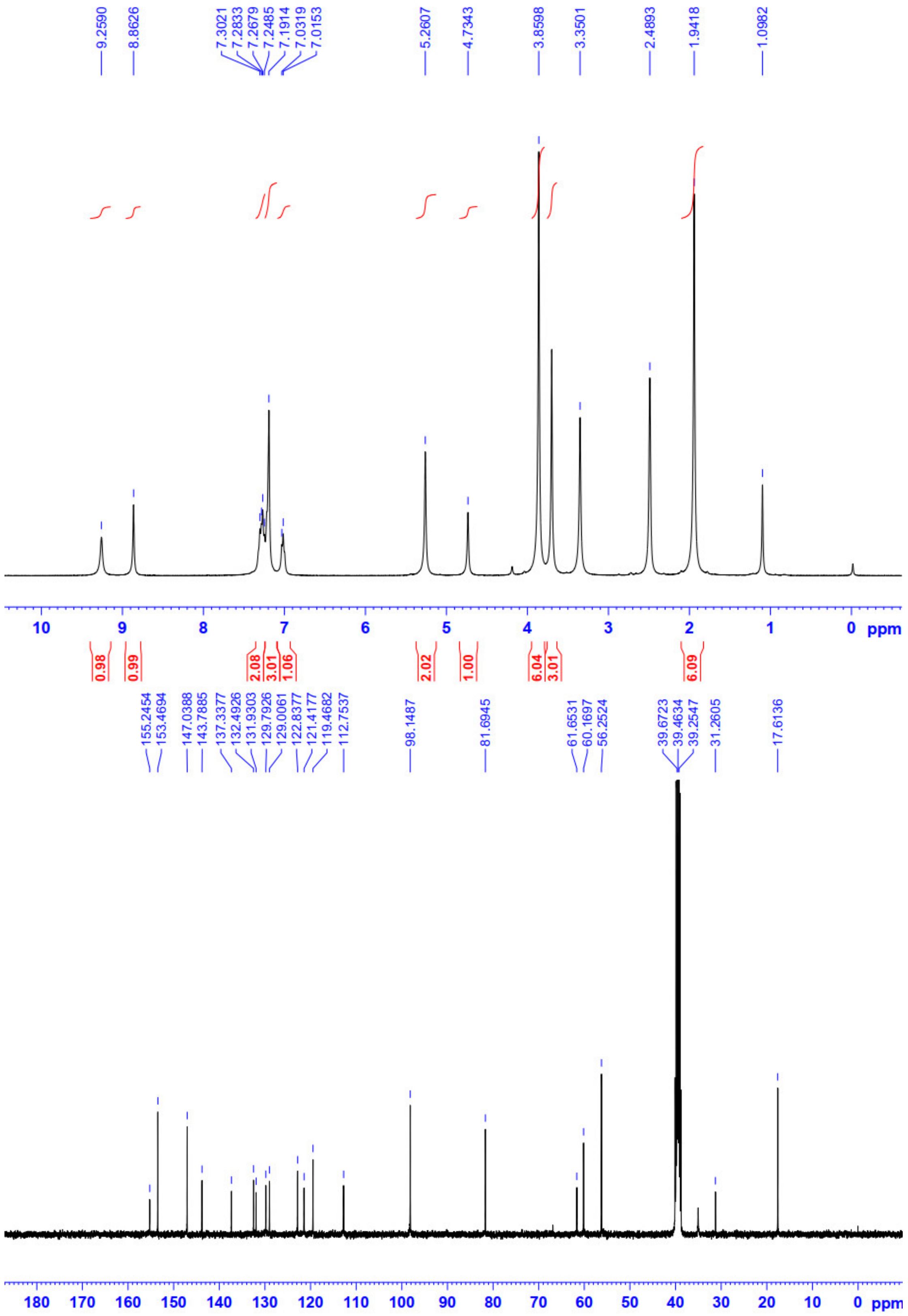

Figure S9. ^{1}H and ^{13}C NMR spectrum of 14bb′ (400 MHz, DMSO-d_6).

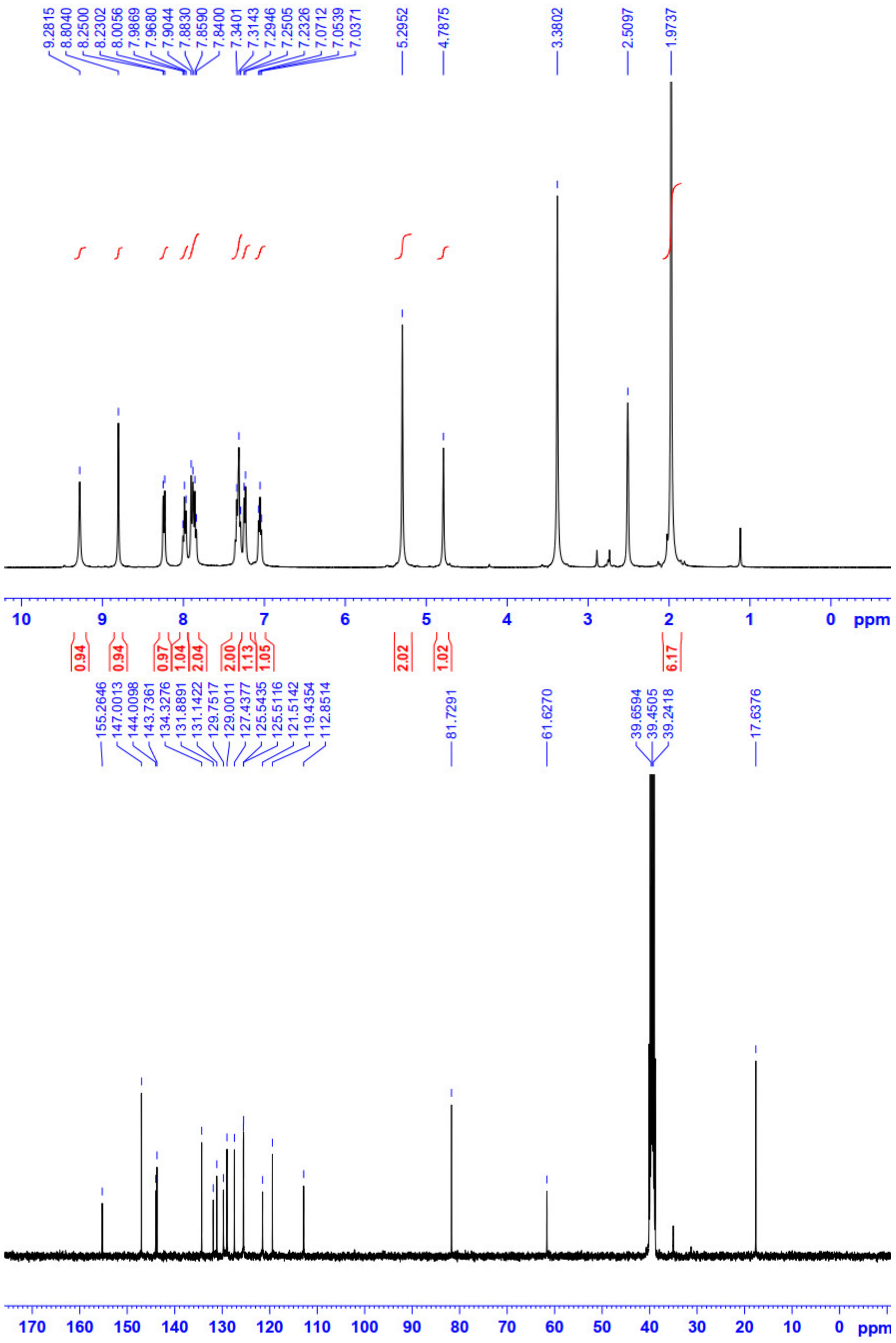

Figure S10. ^{1}H and ^{13}C NMR spectrum of 14bc′ (400 MHz, DMSO-d_6).

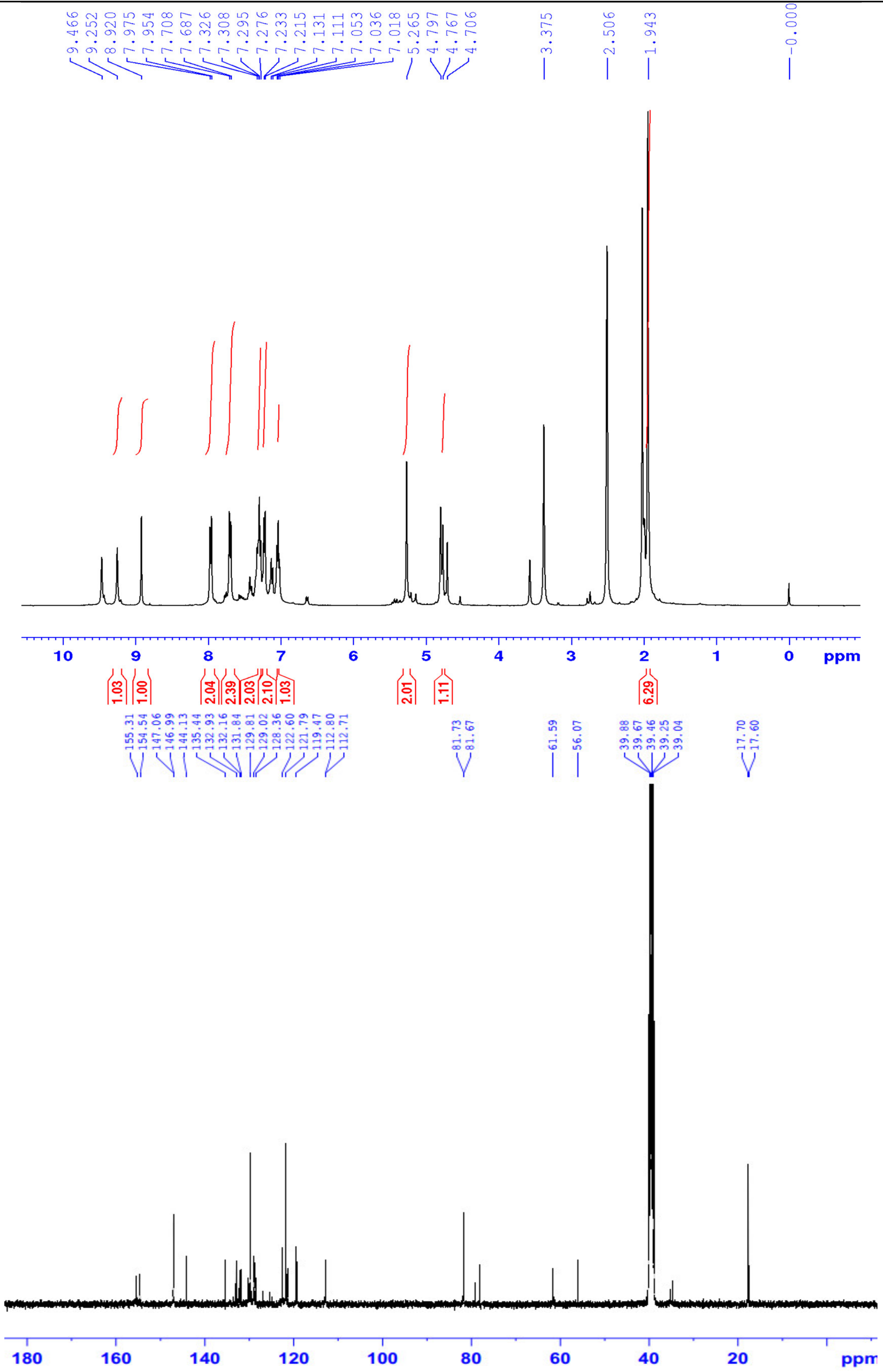

Figure S11. ^{1}H and ^{13}C NMR spectrum of 14bd′ (400 MHz, DMSO-d_6).

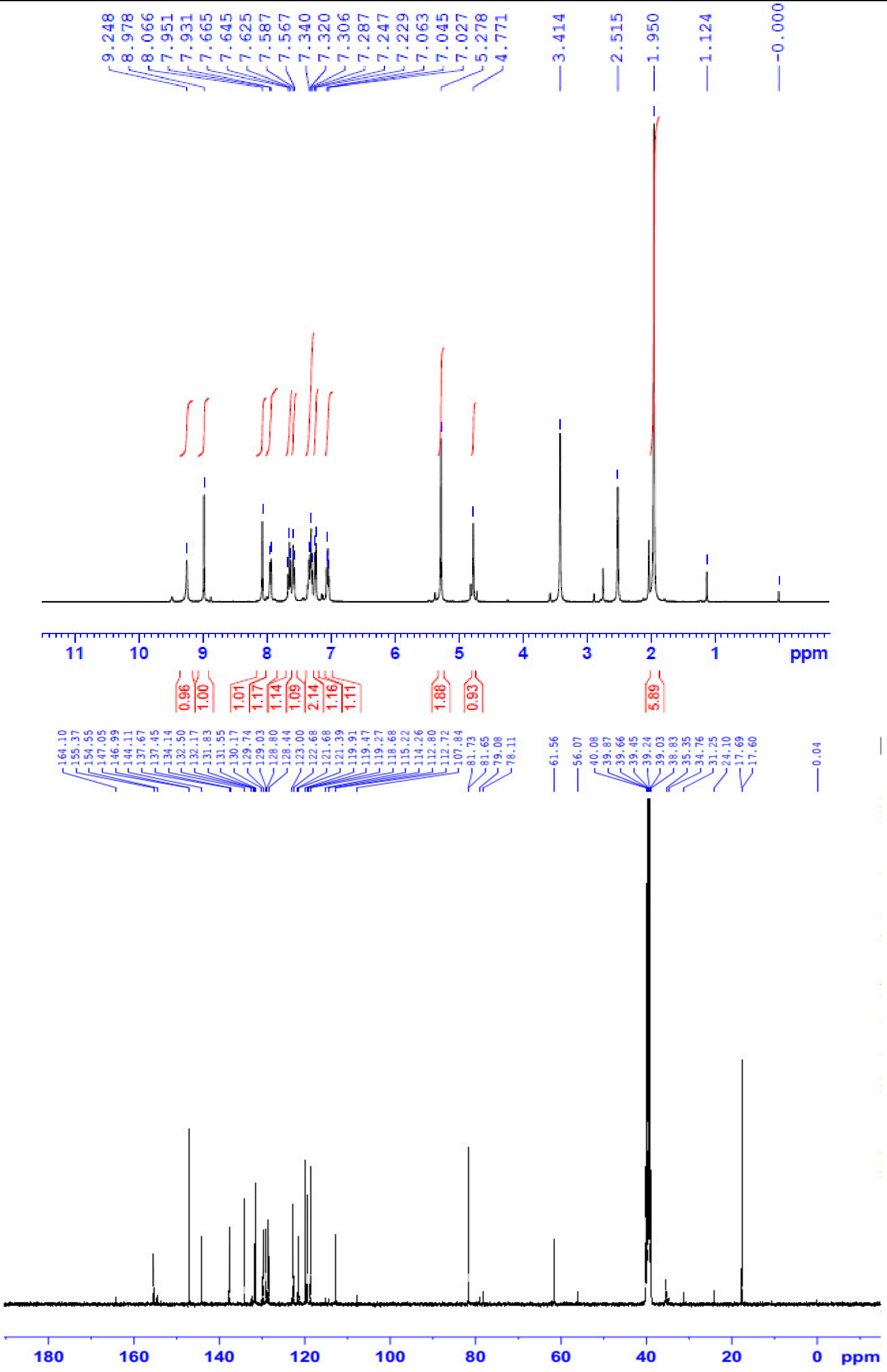

Figure S12. ^{1}H and ^{13}C NMR spectrum of 14be′ (400 MHz, DMSO-d_6).

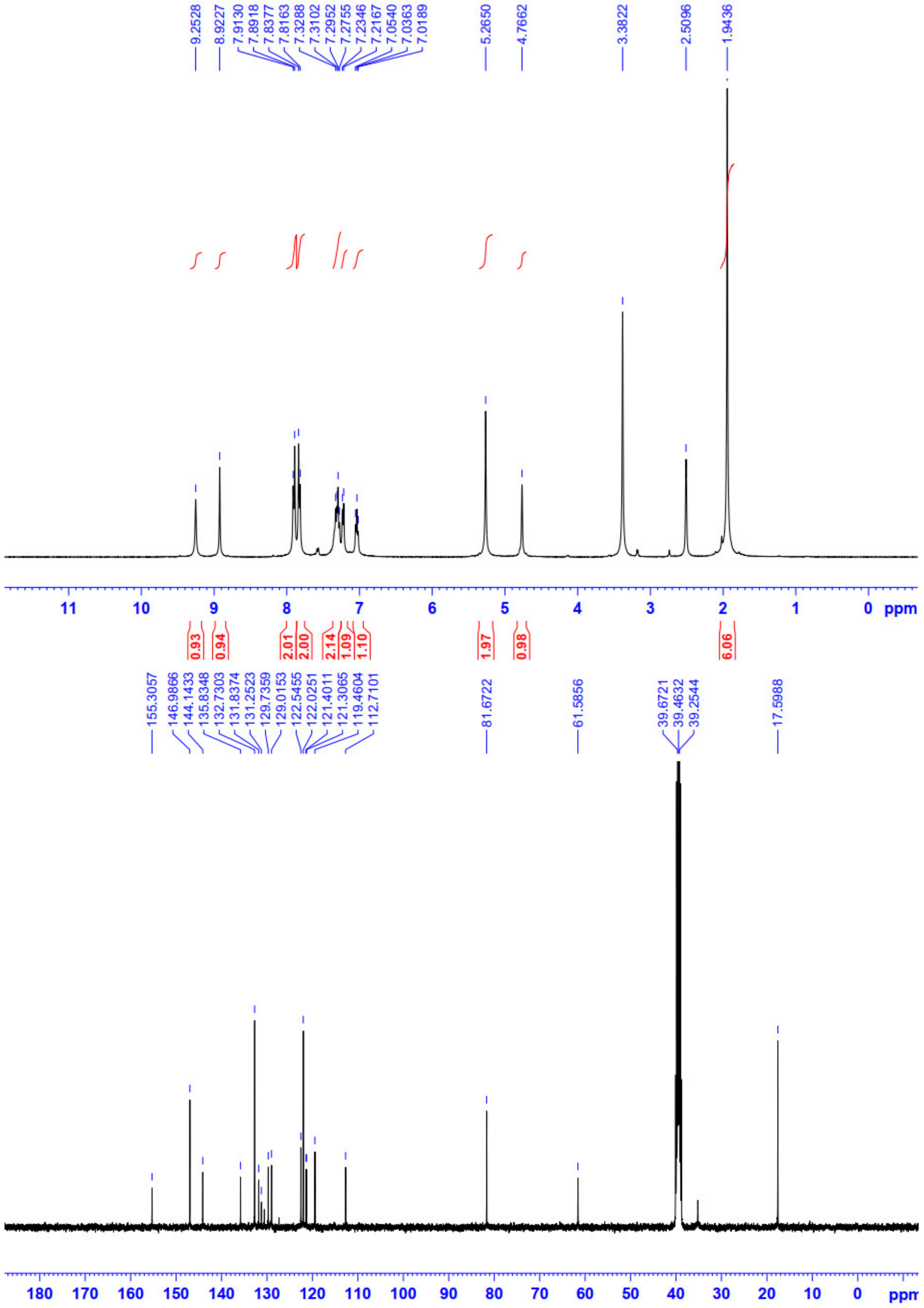

Figure S13. ^{1}H and ^{13}C NMR spectrum of 14bf′ (400 MHz, DMSO-d_6).

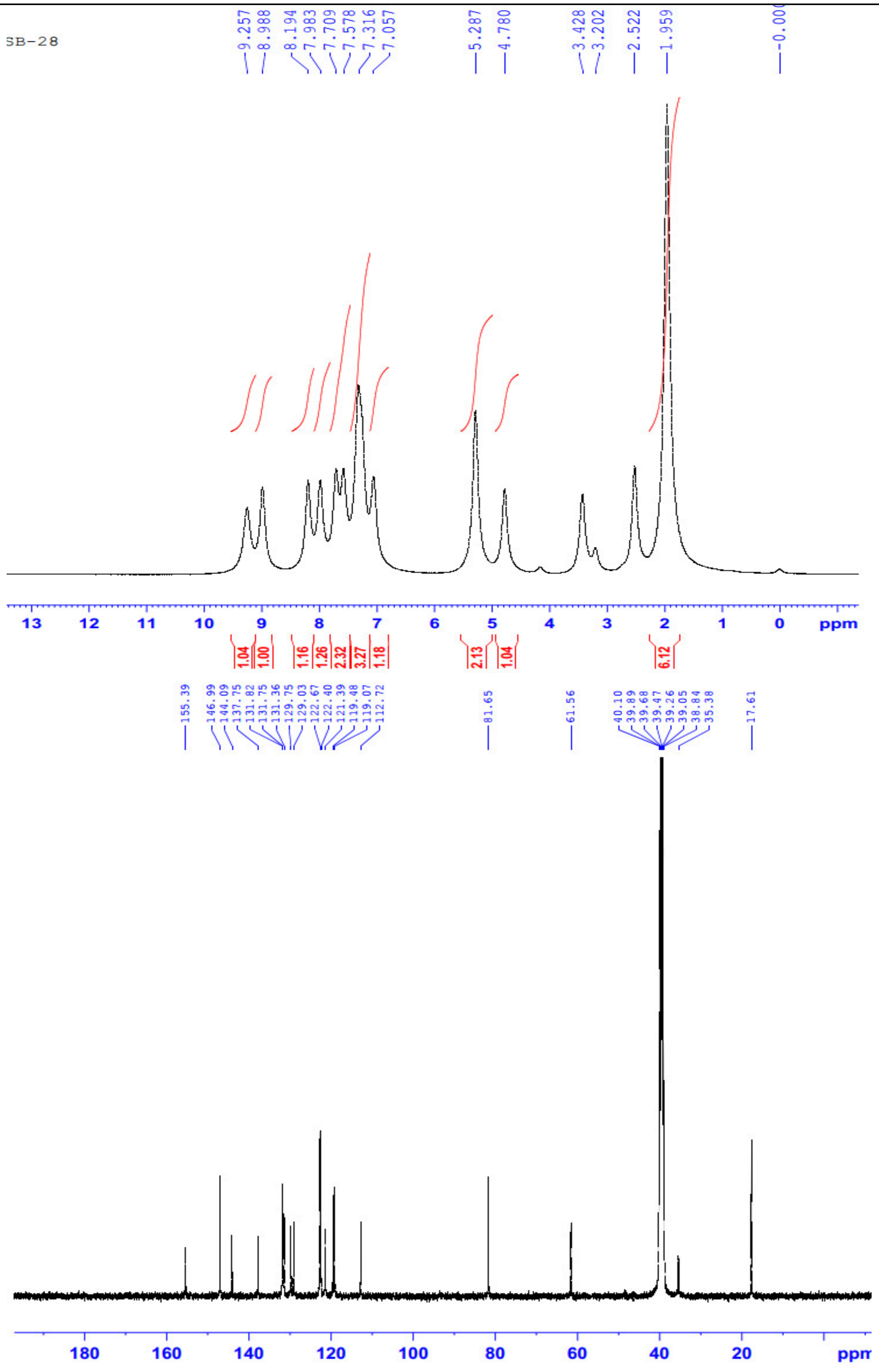

Figure S14. ^{1}H and ^{13}C NMR spectrum of 14bg′ (400 MHz, DMSO-d_6).

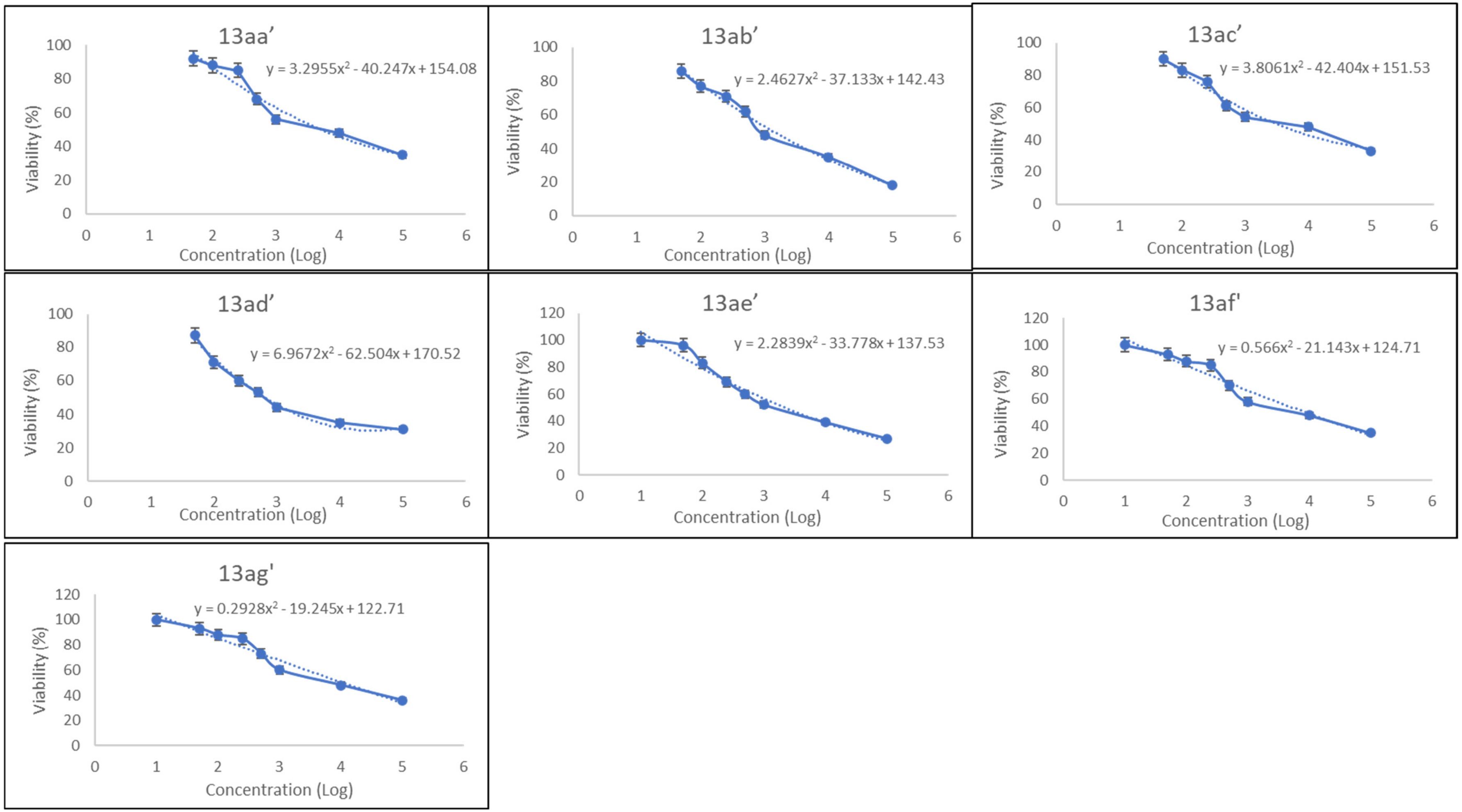

Figure S15. MTT assay graphs of percentage viability for para series compounds (13aa'-13ag').

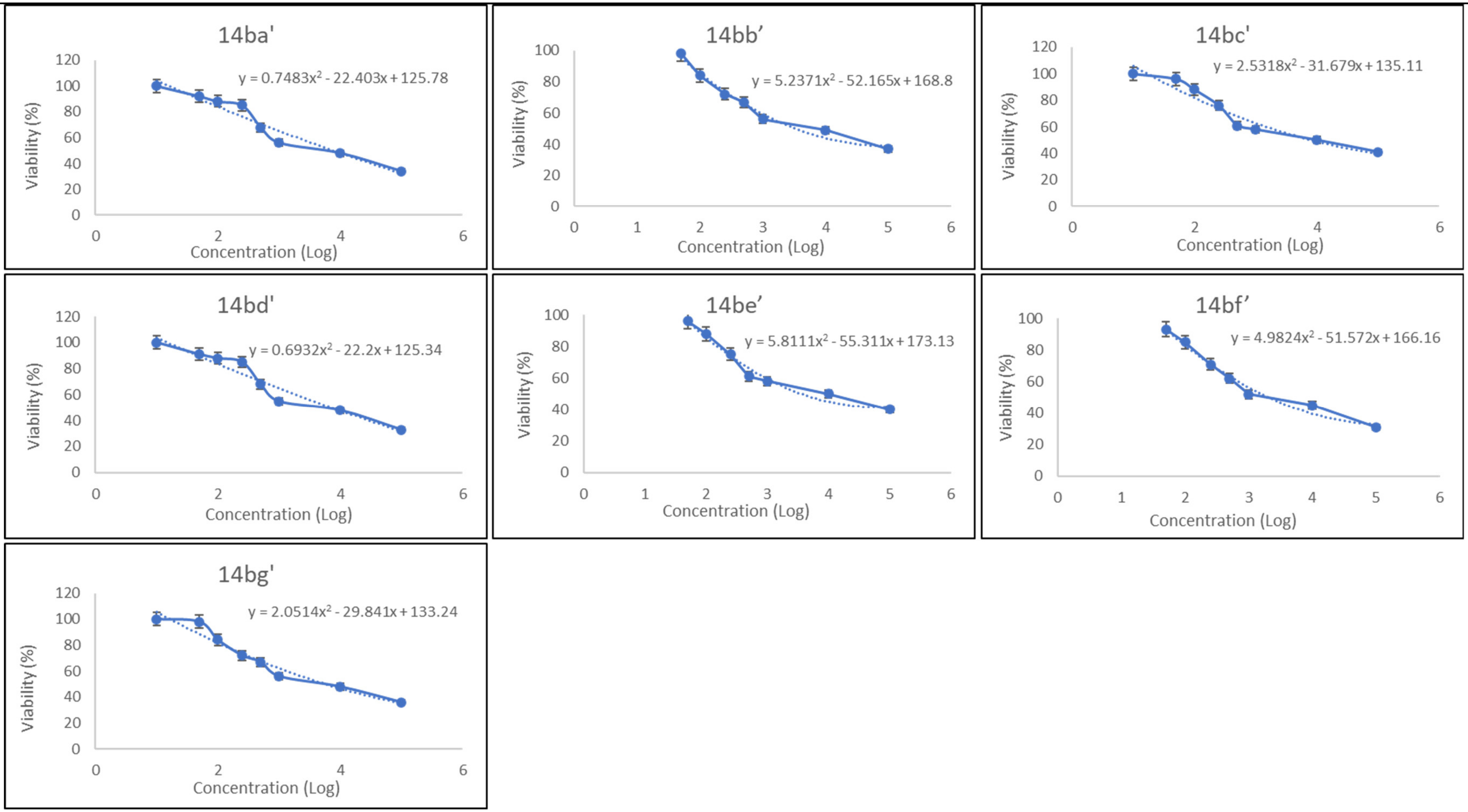

Figure S16. MTT assay graphs of percentage viability for ortho series compounds (14ba'-14bg').

Table S1. Absorbance reading at 570 nm for MTT assay of 13ab' and 13ad' and their IC_{50} value.

Concentration (nM)	Log of concentrations	Absorbance (570nm)						% Viability	
		13ab'			13ad'			13ab'	13ad'
		A	B	C	A	B	C		
	1	0.16	0.181	0.18	0.158	0.183	0.179	100	100
50	1.69897	0.135	0.156	0.158	0.138	0.159	0.156	86	87
100	2	0.124	0.141	0.138	0.113	0.13	0.127	77	71
250	2.39794	0.113	0.129	0.128	0.096	0.109	0.107	71	60
500	2.69897	0.101	0.113	0.11	0.086	0.098	0.097	62	53
1000	3	0.076	0.088	0.087	0.07	0.08	0.079	48	44
10000	4	0.056	0.064	0.062	0.056	0.064	0.062	35	35
100000	5	0.031	0.033	0.03	0.049	0.057	0.056	18	31
	LogIC50							3.145	2.8054
	IC50 (nM)							1396.368	638.8516
